# Low-Temperature Electrolytes for Lithium-Ion Batteries: Current Challenges, Development, and Perspectives

**DOI:** 10.1007/s40820-025-01914-x

**Published:** 2025-09-12

**Authors:** Yang Zhao, Limin Geng, Weijia Meng, Jiaye Ye

**Affiliations:** 1 Xi’an Key Laboratory of Advanced Transport Power Machinery, School of Energy and Electrical Engineering, Chang’an University, Xi’an, 710064 People’s Republic of China; 2 Shaanxi Key Laboratory of New Transportation Energy and Automotive Energy Saving, School of Energy and Electrical Engineering, Chang’an University, Xi’an, 710064 People’s Republic of China; 3 School of Chemistry and Physics, Faculty of Science, Queensland University of Technology, Brisbane, QLD 4001 Australia

**Keywords:** Lithium-ion batteries, Low-temperature electrolyte, Solid electrolyte interphase, Solvation structure, Artificial intelligence-assisted design

## Abstract

Lithium-ion batteries (LIBs), while dominant in energy storage due to high energy density and cycling stability, suffer from severe capacity decay, rate capability degradation, and lithium dendrite formation under low-temperature (LT) operation. Therefore, a more comprehensive and systematic understanding of LIB behavior at LT is urgently required. This review article comprehensively reviews recent advancements in electrolyte engineering strategies aimed at improving the low-temperature operational capabilities of LIBs. The study methodically examines critical performance-limiting mechanisms through fundamental analysis of four primary challenges: insufficient ionic conductivity under cryogenic conditions, kinetically hindered charge transfer processes, Li⁺ transport limitations across the solid-electrolyte interphase (SEI), and uncontrolled lithium dendrite growth. The work elaborates on innovative optimization approaches encompassing lithium salt molecular design with tailored dissociation characteristics, solvent matrix optimization through dielectric constant and viscosity regulation, interfacial engineering additives for constructing low-impedance SEI layers, and gel-polymer composite electrolyte systems. Notably, particular emphasis is placed on emerging machine learning-guided electrolyte formulation strategies that enable high-throughput virtual screening of constituent combinations and prediction of structure–property relationships. These artificial intelligence-assisted rational design frameworks demonstrate significant potential for accelerating the development of next-generation LT electrolytes by establishing quantitative composition-performance correlations through advanced data-driven methodologies.

## Highlights

- Key electrolyte-related factors limiting the low-temperature performance of lithium-ion batteries (LIBs) are analyzed.
- Emerging strategies to enhance the low-temperature performance of LIBs are summarized from the perspectives of electrolyte engineering and artificial intelligence (AI) -assisted design.
- Perspectives and challenges on AI-driven design, advanced characterization, and novel electrolyte systems for low-temperature LIBs.

## Graphical abstract

**Figure:**
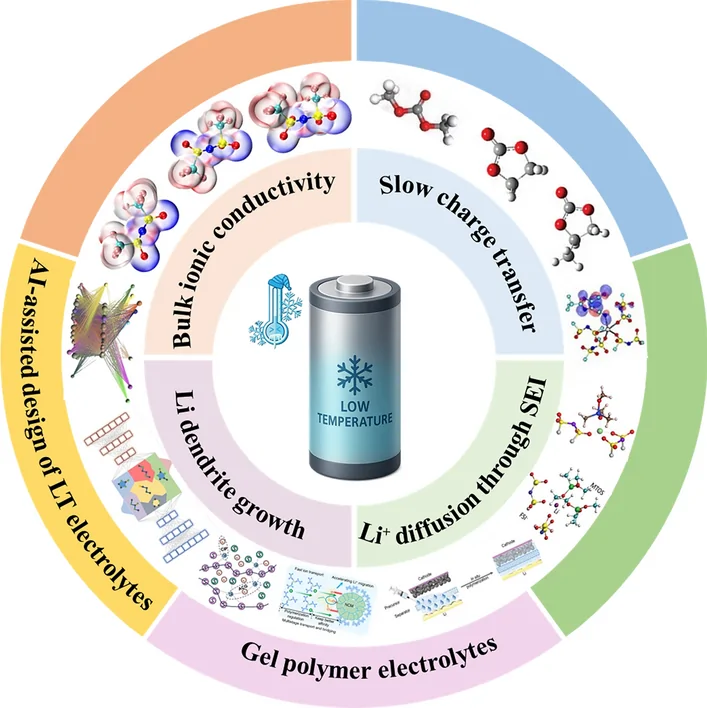

## Introduction

**Fig. 1:**
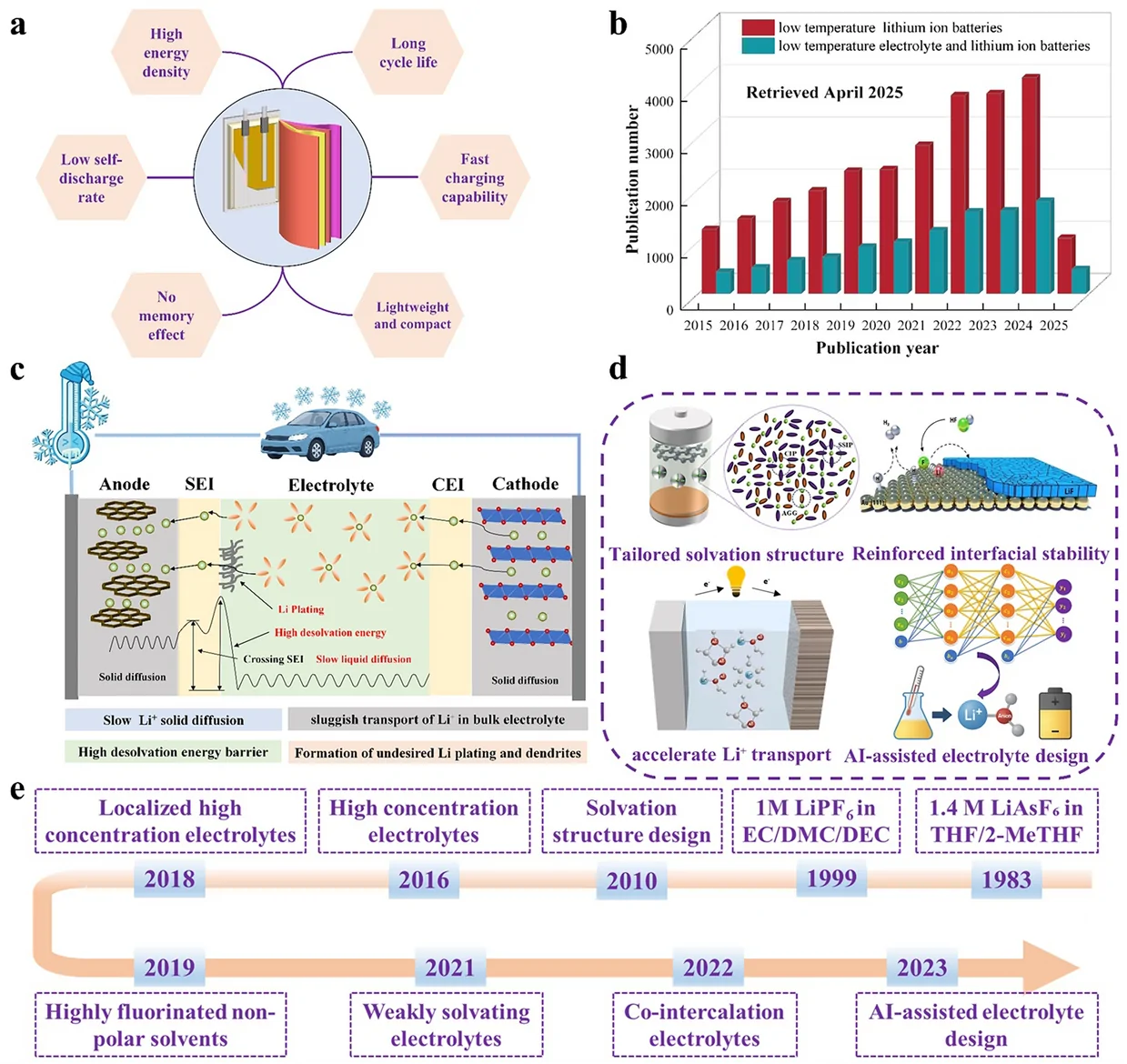
**a** Advantages of LIBs. **b** The number of publications on LT LIBs and LT electrolyte of LIBs from 2015 to 2025 (Data from web of science). **c** Key factors limiting the LT performance of LIBs. **d** Strategies for enhancing the LT performance of LIBs. **e** Milestones in the development of LT electrolytes for LIBs

Driven by the rapid advancement of new energy vehicles, renewable power systems, and portable electronics, the demand for high-performance energy storage systems with high energy density, long lifespan, and rapid charge–discharge capability has been steadily increasing [1–7]. Benefiting from their superior energy density and excellent cycling stability (Fig. 1a), lithium-ion batteries (LIBs) have emerged as the prevailing energy storage technology [8–12]. However, LIB performance and safety deteriorate significantly at subzero temperatures, limiting their use in temperature-sensitive applications, such as satellites, space probes, and submarines [13–16]. Specifically, LIBs typically suffer from rapid capacity degradation, poor rate capability, and sluggish ion transport kinetics at LTs. These issues are often accompanied by safety risks such as lithium (Li) dendrite growth, which severely compromise the cycling life and overall stability of the batteries [17–20]. To improve the performance of LIBs under LT conditions, two main strategies have been proposed. The first entails employing external heating systems to regulate the battery’s temperature, thus alleviating the detrimental effects of cold environments. Nevertheless, this method is typically associated with increased energy consumption, added system complexity, and potential risks in thermal management and operational safety [21, 22].

In contrast, rational molecular design of the electrolyte itself offers a more cost-effective approach to enhancing the LT performance of LIBs. In recent years, the development of LT electrolytes for LIBs has attracted widespread attention, as evidenced by the rapid increase in the number of related publications (Fig. 1b). From the perspective of electrolytes, the key factors limiting the LT performance of LIBs include sluggish Li^+^ transport in the bulk electrolyte, slow solid-phase diffusion of Li⁺, high desolvation energy barriers, and the undesired formation of Li dendrites (Fig. 1c). To rationally design electrolytes that enhance the LT performance of LIBs, several strategies have been proposed (Fig. 1d). Notably, the solid electrolyte interphase (SEI) and cathode electrolyte interphase (CEI) play critical roles in determining electrochemical behavior under LT conditions [3, 23–25]. At LTs, the formation, composition, and physicochemical properties of the SEI and CEI may undergo significant alterations, thereby affecting charge transfer and diffusion processes at the interface. Therefore, elucidating the formation mechanisms and dynamic evolution of SEI/CEI layers under LT conditions is essential for rational interfacial engineering and improving the overall LT performance of LIBs. Furthermore, the rational design of solvation structures plays a pivotal role in improving the performance of electrolytes under LT conditions. By precisely tuning solvent polarity, coordination ability, and molecular architecture, the solvation structure can be optimized to reduce the desolvation energy barrier and enhance interfacial kinetic processes [26–28]. In addition to molecular design strategies, the rapid advancement of artificial intelligence (AI) technologies has opened new avenues for the development of LT electrolytes [29]. Milestones in the development of LT electrolytes for LIBs are illustrated in Fig. 1e. The design of LT electrolytes can be traced back to 1983 [30], when Abraham and colleagues utilized tetrahydrofuran (THF) and 2-methyltetrahydrofuran (2MeTHF) to formulate an electrolyte aimed at enhancing the LT performance of lithium-titanium disulfide cells. An electrolyte based on a ternary mixed solvent system of ethylene carbonate (EC), dimethyl carbonate (DMC), and diethyl carbonate (DEC) with a volume ratio of 1:1:1 was first reported in 1999 [31]. Under LT conditions, it exhibited superior ionic conductivity and film-forming characteristics compared to binary systems composed of EC/DMC (3:7 v/v) or EC/DEC (3:7 v/v). Subsequently, numerous electrolytes based on ternary or quaternary mixed solvent systems have been proposed to further enhance the LT performance of LIBs [32–34]. In 2010, Yang et al. investigated the solvation structure of lithium hexafluorophosphate (LiPF₆) in a ternary mixed solvent system consisting of EC, DMC, and DEC [35]. Their experimental results confirmed that EC exhibits a stronger coordination ability with Li⁺ compared to DEC and DMC. Increasing attention has been devoted to enhancing the LT performance of LIBs by tuning the solvation structure of the electrolyte. For instance, propylene carbonate (PC), employed as a functional dopant, can effectively modulate the solvation structure dominated by 2,2,2-trifluoroethyl methyl carbonate through dipole–dipole interactions and subtle microsolvation competition [36]. As a result, the electrolyte enables stable operation at − 60 °C. Due to their unique ability to regulate solvation structures, high concentration electrolytes (HCEs) [37] have attracted widespread attention in recent years. Subsequently, localized high concentration electrolytes (LHCEs) [38], developed by modifying these systems, have also been extensively employed to enhance the LT performance of LIBs. Xie et al. reported a diluted high-concentration electrolyte (DHCE) based on cyclopentylmethyl ether (CPME), which enabled the stable operation of lithium metal batteries (LMB) at − 60 °C [39]. This electrolyte promoted the formation of ion clusters, thereby facilitating anion-dominated interfacial chemistry, enhancing the interfacial compatibility of the lithium metal anode (LMA), and significantly accelerating the desolvation kinetics of Li⁺. In addition, a DHCE incorporating ethoxy (pentafluoro) cyclotriphosphazene (PFPN) as a multifunctional diluent has been reported [40]. This DHCE forms an anion-dominated solvation structure that enhances the thermodynamic stability of the electrolyte. Through synergistic interactions with anions, PFPN promotes the formation of a LiF-rich SEI on the anode surface, effectively suppressing the continuous decomposition of the electrolyte. This DHCE not only demonstrates high safety but also enables stable operation at − 60 °C. Weakly solvating electrolytes (WSEs) [41] have further improved the LT performance of LIBs. For example, in WSEs based on the weakly solvating solvent ethyl trifluoroacetate (ETFA), a subtle competitive microsolvation effect leads to the formation of a loose Li⁺ coordination structure [42]. This structure combines moderate Li⁺ affinity with relatively high ionic conductivity. As a result, the electrolyte enables reversible charging and discharging of LIBs at − 40 ℃. With the rapid development of AI technologies, their powerful capabilities in data mining and nonlinear modeling are increasingly being applied to the design and optimization of LT electrolytes [43]. This approach not only facilitates a systematic analysis of the complex structure–performance relationships among multicomponent systems–such as solvents, Li salts, and additives–but also significantly improves the efficiency and predictive accuracy of low-temperature electrolyte development. Consequently, AI offers an effective means to overcome the limitations of traditional trial-and-error methods, enabling more efficient molecular screening and formulation optimization of electrolytes. Recently, several reviews have summarized the research progress of LT LIBs in terms of material systems and electrolyte regulation [44–46]. However, a comprehensive overview of molecular design strategies for LT electrolytes, as well as AI-assisted electrolyte design approaches, is still lacking.

**Fig. 2:**
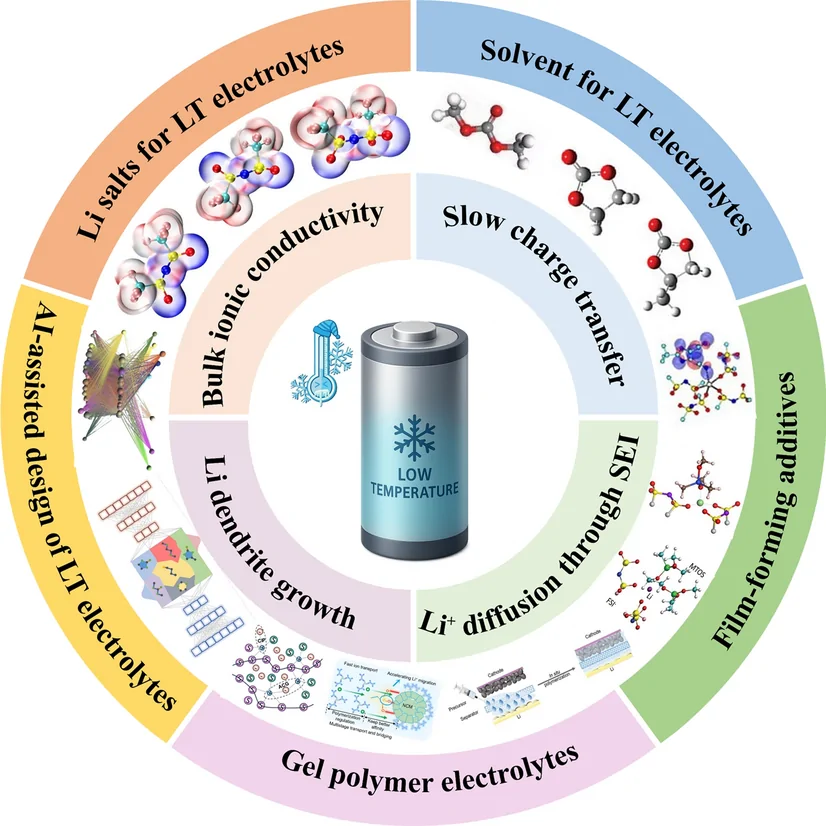
Design strategies for LT electrolytes in LIBs

In this review, the key mechanisms affecting the LT performance of LIBs are systematically summarized by analyzing the migration kinetics of Li ions, the compositional evolution and structural characteristics of the SEI, and the Li deposition behavior at LTs (Fig. 2). Subsequently, recent advances in optimization strategies aimed at enhancing the LT performance of LIBs are presented, including the selection of Li salts, solvent system optimization, and interfacial engineering. In particular, AI-assisted strategies for the design of LT electrolytes are highlighted. Based on these strategies, the interrelationship among Li⁺ desolvation energy barriers, solvation structures, and SEI formation with the LT performance of LIBs is systematically discussed. The importance of tailoring solvation structures for the rational design of LT electrolytes is emphasized. Finally, the future development trends of LT electrolytes are discussed, aiming to promote the advancement of high-performance LIBs operating in subzero environments.

## Challenges of LIBs at LTs

LIBs encounter a series of challenges under LT conditions, which significantly compromise their electrochemical performance and safety. Therefore, it is imperative to conduct an in-depth analysis of the key factors influencing the LT performance of LIBs. These factors can be categorized as follows: (1) Limitation of bulk ionic conductivity. (2) Slow charge transfer. (3) Diffusion of Li ions through SEI. (4) Acceleration of Li plating and Li dendrite growth. The following sections will discuss each of these aspects in detail.

### Limitation of Bulk Ionic Conductivity

**Fig. 3:**
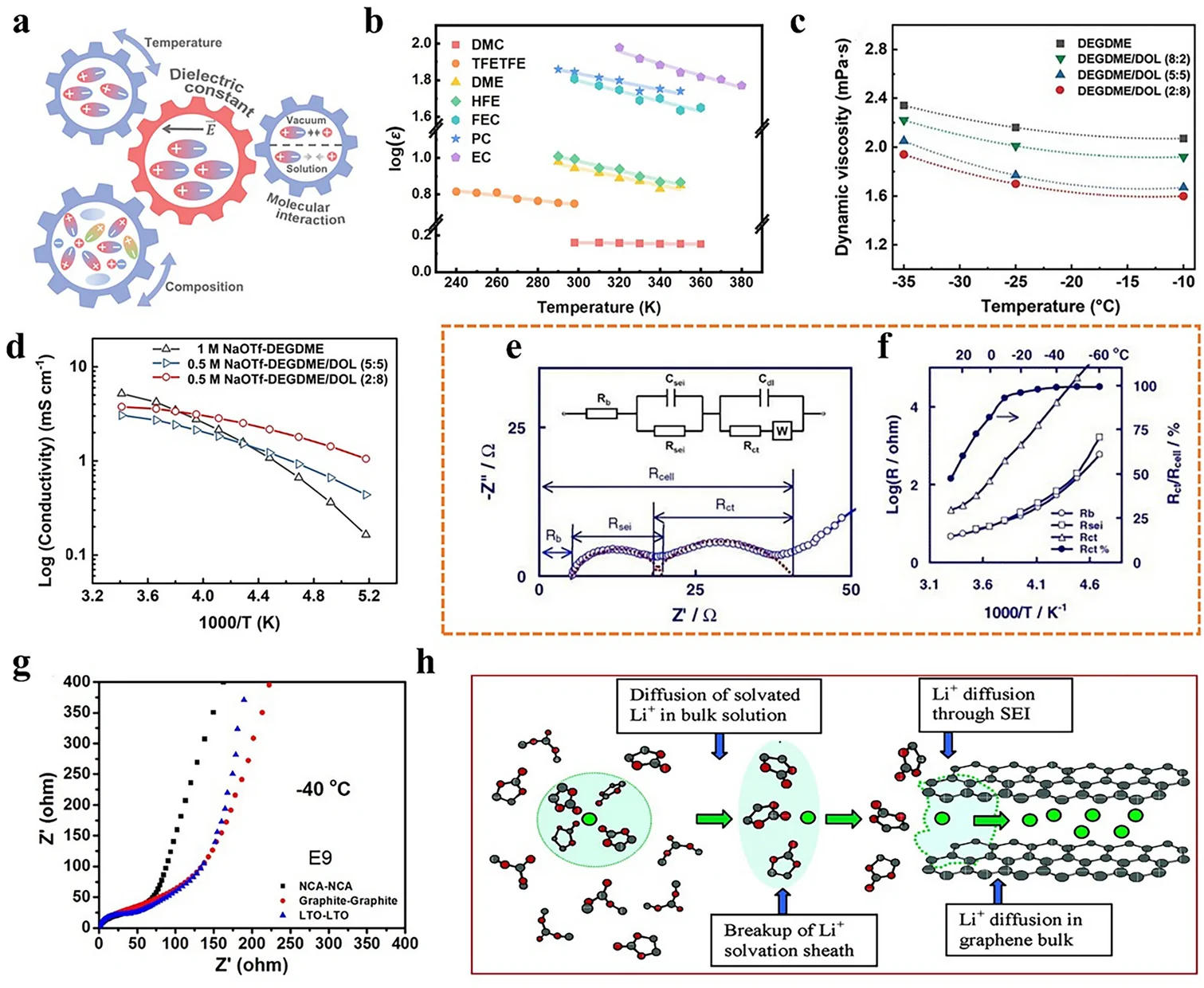
**a** Relations among temperature, electrolyte composition, DC, and molecular-level interactions [47]. **b** Variation of DC of various solvents with temperature [47]. **c** Dynamic viscosity of electrolytes at different temperatures [50]. **d** Ionic conductivity of electrolytes at different temperatures [50]. **e** Typical EIS of the Li-ion cell and the equivalent circuit used to fit the EIS [53]. **f** Temperature dependences of the *R*_b_, *R*_SEI_ and *R*_ct_ of the Li-ion cell at 3.87 V [53]. **g** Comparison of EIS results for symmetric cells of NCA || NCA, Gr || Gr and LTO || LTO at − 40 °C [61]. **h** Schematic descriptions of the post-SEI journey of a solvated Li^+^ from solution bulk to graphene interior [62]

The electrolyte plays a major role in transporting Li ions inside LIBs. Ionic conductivity is a key characteristic of the electrolyte, which determines the migration efficiency of Li ions in the electrolyte, and thus affects the LT performance of the battery. The dielectric constant (DC) is an important physical property of liquids, commonly used to characterize solvent polarity, and is widely applied in evaluating solubility, viscosity, and ionic conductivity. Notably, as the temperature decreases, the DC of the solvent tends to increase, which may be attributed to the reduced molecular mobility (Fig. 3a, b) [47]. For electrolytes of conventional concentration, ion transport is a coupled process involving ion diffusion and the reorganization of solvent molecules (or polymer chain segments). Consequently, the temperature dependence of ionic conductivity deviates from the linear Arrhenius relationship, instead exhibiting a nonlinear Vogel–Tammann–Fulcher relationship [48, 49]:

1 $$ \sigma = AT^{ - 1/2} \exp ( - B/R(T - T_{0} )) $$

where *σ* is the ionic conductivity, *A* is the pre-exponential factor, *B* represents the pseudo-activation energy associated with the structural reorganization of solvent molecules (or polymer segments), *T* is the ambient temperature, and *T*_0_ is the temperature at which the configurational entropy of solvent molecules or polymer segments is zero (which can be regarded as the freezing point of the electrolyte). When the temperature is lower than *T*_0_, the solvent molecules or polymer segments can no longer undergo structural reorganization to assist ion transport, causing the ionic conductivity of the electrolyte to drop precipitously, and the LIBs will be unable to charge and discharge normally.

The solvent–solvent interactions are more pronounced at LTs, which significantly hinders the movement of solvent molecules, resulting in a significant increase in viscosity (Fig. 3c) [50]. For instance, at room temperature, conventional electrolytes based on ethylene carbonate (EC) typically maintain an EC content between 30 and 50%, resulting in relatively high ionic conductivity (approximately 10 m S cm^−1^), which is considered viable [51]. However, when the temperature drops to 0 °C, the viscosity of EC-containing electrolytes increases dramatically. This rise in viscosity impedes the transport of Li^+^ within the bulk electrolyte, leading to a marked decline in ionic conductivity (Fig. 3d) [50]. As the temperature decreases, ion–solvent and ion–ion interactions progressively intensify. This enhanced interaction causes solvent molecules and counterions to more closely surround cations, forming larger solvated ion clusters. In electrolytes, charge carriers are primarily transported in the form of these solvated ions. Consequently, the formation of abundant solvated ion clusters inevitably hinders the efficient transport of ions, leading to a rapid decline in ionic conductivity at LTs.

The intensification of solvent–solvent and ion-ion interactions further diminishes ionic conductivity by altering the properties of the Li salt. Specifically, on the one hand, the interaction between solvents gradually increases, and the solvent's dissolving ability also decreases, which reduces the solubility of Li salts. On the other hand, the interaction between cations and anions also increases, prompting Li salt crystals to begin to precipitate in a LT environment. This process leads to a further decrease in the concentration of carrier ions in the electrolyte, which inevitably causes a decrease in ionic conductivity [52]. When the electrolyte can effectively transport Li⁺ between electrodes, ionic conductivity is not the only factor affecting the low temperature performance of LIBs [13]. The stability of the liquid phase at LTs is also an important factor. When the temperature drops below the freezing point of the electrolyte, the electrolyte freezes and almost completely loses the ability to transport Li⁺, causing the LIBs to fail.

### Slow Charge Transfer

Extensive research has shown that the sluggish charge transfer rate is widely regarded as a critical factor limiting the LT kinetic performance of LIBs. Electrochemical impedance spectroscopy (EIS) is a key technique used to study charge transfer processes. In general, the total impedance of a LIBs comprises the ohmic resistance (*R*_b_), the SEI resistance (*R*_SEI_), and the charge transfer resistance (*R*_ct_) [53]. Among these, the charge transfer resistance *R*_ct_ is considered the most critical factor influencing the LT performance of LIBs [54]. The *R*_ct_ is commonly used to quantify the difficulty of the charge transfer process. According to the Arrhenius equation and the Butler–Volmer equation, *R*_ct_ is directly influenced by the activation energy (*E*_a_) associated with charge transfer. A higher *E*_a_ results in a larger *R*_ct_, which reduces the charge transfer rate and adversely affects battery performance at LTs. *R*_ct_ is described by the Arrhenius equation as follows:

2 $$ \frac{1}{{R_{{{\mathrm{ct}}}} }} = A\exp \left( {\frac{{ - E_{{\mathrm{a}}} }}{RT}} \right) $$

where *R* denotes the universal gas constant. A higher *R*_ct_ value indicates sluggish charge transfer reaction kinetics.

The desolvation resistance (*R*_desolvation_), which represents the resistance encountered by Li^+^ during the desolvation process before entering the material's interlayers, constitutes the primary component of *R*_ct_. The *R*_ct_ usually dominates the total impedance of LIBs at LTs [55]. Zhang and his collaborators have shown that at LTs, the increase in *R*_ct_ is more obvious than the *R*_b_ and the *R*_SEI_ (Fig. 3e) [53]. At 20 °C, *R*_ct_ accounts for less than 40% of the total impedance, and below − 20 °C, *R*_ct_ is almost equal to the total resistance. The tested samples employed an electrolyte of 1.0 M LiPF_6_ in EC/ethyl methyl carbonate (EMC) (3:7 by weight), with standard graphite (Gr) as the anode material and a lithium nickel-based mixed oxide as the cathode material. Recent studies have also shown that the *R*_ct_ increases rapidly as the temperature decreases. For example, Yin et al. investigated the impedance behavior of LiFePO_4_ (LFP) || Li cells using EIS under different temperatures, with an electrolyte formulation of 1.0 M LiPF₆ in EC/DMC/EMC (1:1:3 by volume) [56]. Their results revealed that, regardless of charge or discharge state, the charge transfer resistance *R*_ct_ was significantly higher than the *R*_b_ and *R*_SEI_ as the temperature decreased. During discharge at 20 °C, the *R*_ct_ was 389.5 Ω cm^2^, which increased dramatically to 7076 Ω cm^2^ at − 20 °C and 31,930 Ω cm^2^ at − 40 °C. Similarly, during charging, the *R*_ct_ rose from 430 Ω cm^2^ at 20 °C to 2124 Ω cm^2^ at − 20 °C and 19,530 Ω cm^2^ at − 40 °C. In addition, for Li [Li_0.1_Al_0.1_Mn_1.8_] O_4_ (LAMO) || LAMO symmetric cells, the charge transfer resistance *R*_ct_ measured at 25 °C was 70 Ω cm^2^, which increased significantly to 400 Ω cm^2^ at − 15 °C [57].

Charge transfer is not a singular process, but a coupling of multiple kinetic processes [58]. In terms of frequency response, it typically correlates with the impedance in the mid-frequency band, which overlaps with the transfer process of Li^+^ in the SEI [59]. Therefore, it is necessary to analyze the key factors that contribute to the increase in *R*_ct_ at LTs. As shown in Eq. (2), the charge transfer resistance is determined by the temperature of the charge transfer process and the activation energy (*E*_a_). Abe et al. [60] found that the *E*_a_ for the transfer of solvated Li ions at the graphite–electrolyte interface is 25 kJ mol^−1^, which is significantly lower than the 53–59 kJ mol^−1^ required for the removal of the solvent and formation of bare Li ions (Fig. 3f). This difference in activation energies highlights the difficulty of Li^+^ desolvation. The research of Li et al. further showed that the desolvation process of Li^+^ is not only the dominant factor in charge transfer but also determines the LT performance of LIBs [61]. They conducted EIS tests at − 40 °C on three assembled symmetric cells (LiNi_0.8_Co_0.15_Al_0.05_O_2_(NCA) || NCA, Gr || Gr, Li_4_Ti_5_O_12_(LTO) || LTO). All three batteries employed the same carbonate-based electrolyte, which eliminated the influence of varying electrode materials and solvation structures. Experimental results revealed that, despite the differences in interfacial chemistries and material properties among the three cells, their EIS spectra at − 40 °C exhibited similar characteristics. (Fig. 3g). They concluded that the most plausible explanation was that the Li⁺ desolvation process accounted for the majority of the impedance at − 40 °C. On this basis, Xu et al. studied the activation energy of the mid-frequency impedance of Gr electrodes by EIS to distinguish the contribution of the charge transfer process and the Li^+^ transport process in SEI to the mid-frequency impedance [62]. They found that the activation energy *E*_a_ of the mid-frequency impedance was about 60–70 kJ mol^−1^, of which the *E*_a_ of the charge transfer process was about 50 kJ mol^−1^ and the *E*_a_ of the Li^+^ transport in SEI was about 10–20 kJ mol^−1^, which showed that the charge transfer process had the main contribution to the mid-frequency impedance (Fig. 3h). In addition, they also found that the activation energy of charge transfer is related to the solvent in the electrolyte. The *E*_a_ of conventional carbonate solvents is about 50 kJ mol^−1^, compared with ether solvents, which have lower charge transfer activation energy (the *E*_a_ of THF is about 40 kJ mol^−1^). This also shows that the desolvation of Li^+^ is the dominant factor in the charge transfer process.

### Diffusion of Li Ions Through SEI

**Fig. 4:**
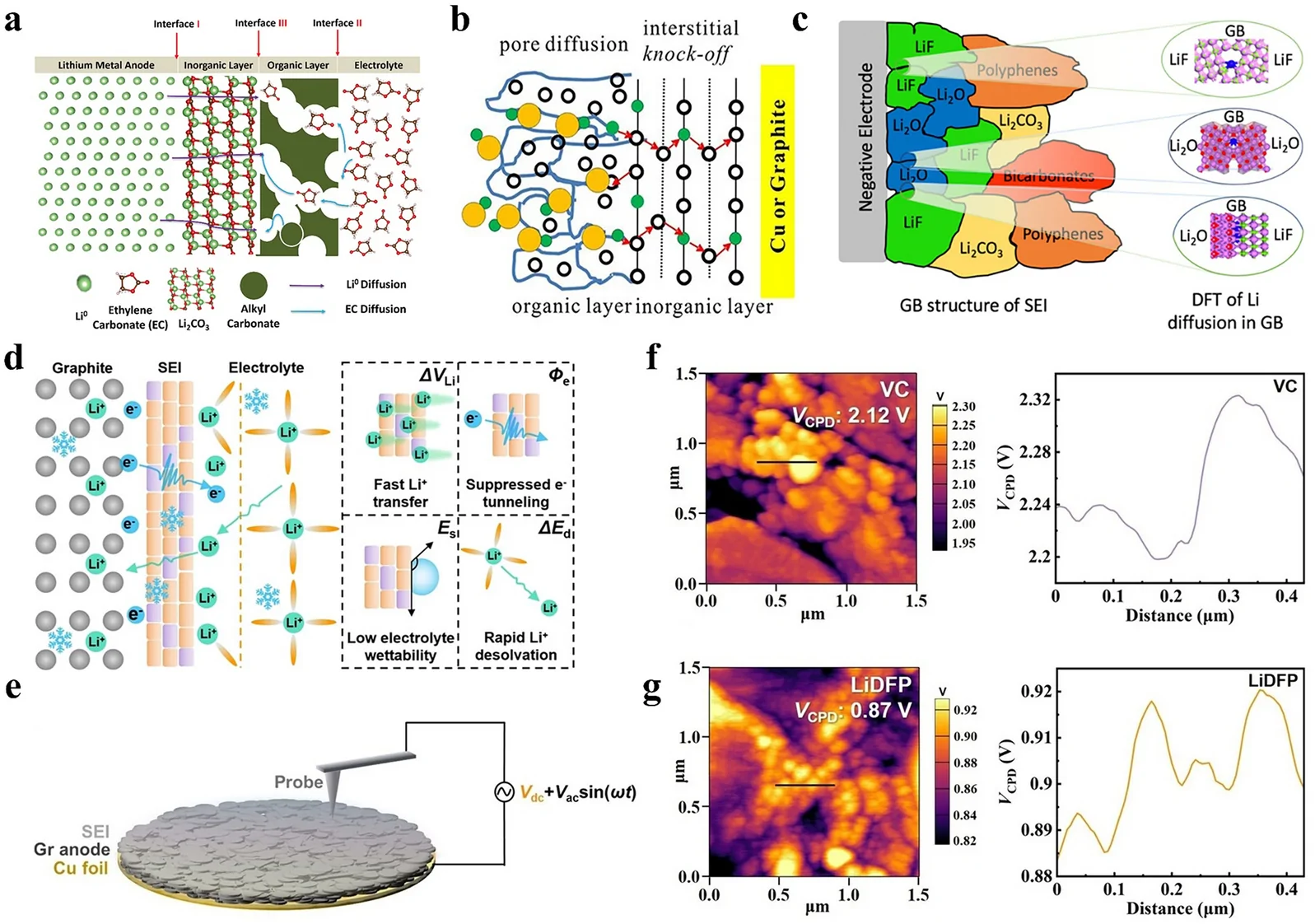
**a** Schematic illustration of SEI structure and growth mechanism [63]. **b** Li⁺ diffusion in the SEI [70]. **c** Grain boundaries structure of SEI [74]. **d** Schematic illustration of the construction of S_SEI_ as a thermodynamic descriptor [80]. **e** Schematic illustration of KPFM measurement [80]. *V*_CPD_ mappings in KPFM images and the representative *V*_CPD_ -distance curves corresponding to the black scanning line for the SEI layer derived in **f** VC and **g** LiDFP electrolytes [80]

The operation of a LIBs involves the desolvation of Li^+^ and its subsequent diffusion across the SEI and CEI membranes. The SEI is a composite phase formed at the electrode/electrolyte interface, primarily consisting of decomposition products of the electrolyte on the electrode surface (Fig. 4a) [63]. It effectively isolates the electrode from the electrolyte, preventing the continuous occurrence of side reactions [64]. Enhancing the diffusion rate of Li^+^ within the SEI is crucial for improving the performance of LIBs at LTs, as the diffusion of Li^+^ in the SEI is significantly more challenging compared to its diffusion in the liquid electrolyte [65]. The Gr anode has been shown to exhibit a relatively low energy barrier (4.9 kJ mol^−1^) for bulk Li⁺ diffusion [66], indicating that the impact of temperature reduction on ion transport during the intercalation process is relatively minor. In contrast, the diffusion of Li⁺ across the SEI requires a significantly higher activation energy (14.4 kJ mol^−1^) [63], making this process the rate-determining step during LT cycling.

The diffusion mechanism of Li^+^ within the SEI is a complex process influenced by multiple factors, including the SEI’s structure, chemical composition, and temperature. A deeper understanding of the diffusion mechanism of Li^+^ within the SEI is crucial for enhancing the LT performance of LIBs. However, due to the limitations of characterization techniques, the precise diffusion mechanism of Li^+^ in the SEI remains a subject of debate. The diffusion of Li^+^ within the SEI may involve two distinct mechanisms. The outer layer of the SEI is relatively porous, rich in organic phases and certain inorganic components, and exhibits a high porosity. As a result, Li^+^ primarily diffuses through the gaps in the outer layer in the form of point defects [67]. The inner layer of the SEI is primarily composed of inorganic compounds such as Li_2_CO_3_, LiF, Li_2_O, forming a highly dense structure. Among them, LiF has excellent mechanical strength and significant Li dendrite suppression ability [68, 69]. In this region, Li^+^ migrates mainly through a knock-off mechanism within the crystalline lattices of Li_2_CO_3_ and other inorganic phases, rather than simple vacancy hopping **(**Fig. 4b**)** [70].

The composition and structure of the SEI are highly complex, and several studies have summarized the key components and structural characteristics of the SEI [64, 71, 72]. The SEI membrane forms after the electrolyte decomposes during the initial cycles, and its composition and structure are directly determined by the types of ions and molecules present in the electrolyte. Zhang et al. discovered that the SEI membrane consists of a dense inorganic inner layer and an organic outer layer permeated by the electrolyte [73]. An ideal SEI membrane should be rich in inorganic components while maintaining a thin and compact structure. Increasing the proportion of inorganic components in the SEI can enhance Li^+^ diffusion, likely because Li^+^ diffuses more rapidly in the inorganic layer compared to the organic layer. First-principles calculations indicate that Li^+^ exhibits a higher diffusion rate along the grain boundaries of inorganic SEI components such as Li_2_O, LiF, and Li_2_CO_3_ (Fig. 4c) [74]. Studies have shown that the interaction between Li_2_CO_3_ and LiF can facilitate the accumulation of space charges at their interface, thereby increasing the concentration of ionic charge carriers and effectively enhancing Li^+^ transport efficiency [75]. Li^+^ has a faster diffusion rate in the SEI containing the inorganic component Li nitride (Li_3_N), and the effect is particularly significant at LTs [72, 76]. This phenomenon further confirms that Li^+^ diffuses more rapidly within the inorganic layer. Therefore, an SEI membrane rich in inorganic components can significantly enhance Li^+^ mobility, making it an ideal electrode–electrolyte interface for optimizing the LT performance of LIBs. Optimizing the solvation structure can regulate the inorganic/organic composition ratio, thickness, density, and porosity of the SEI film, thereby enhancing the diffusion characteristics of Li⁺. For example, the SEI formed in an electrolyte consisting of 0.6 M Lithium bis (fluorosulfonyl) imide (LiFSI) + 0.2 M Lithium difluoro (oxalato) borate (LiDFOB) dissolved in 1,3-Dioxacyclohexane (1,3-DIOX) is primarily composed of LiF, B-containing compounds, and a small amount of Li_2_CO_3_. In contrast, the SEI generated in an electrolyte with 0.8 M LiFSI dissolved in 1,3-DIOX mainly consists of Li_2_CO_3_ and organic compounds, with a minor presence of LiF [77].

LTs can affect the composition and structure of the SEI, thereby influencing the migration rate of Li^+^ within it. Studies have shown that the desolvation of Li^+^ is the primary source of high activation energy, while the diffusion of Li^+^ within the SEI accounts for only a small proportion [61]. However, this does not imply that the impact of Li^+^ diffusion within the SEI on LT performance can be overlooked. On one hand, after prolonged cycling, the SEI becomes thicker, making the transport of Li^+^ within the SEI more difficult, and this phenomenon may be more pronounced at LTs. On the other hand, the desolvation process and the diffusion of Li^+^ within the SEI interact with each other. When diffusion occurs in the relatively loose outer layer of the SEI, Li ions may gradually remove solvent molecules, suggesting that the structure of the SEI could potentially influence the desolvation process [78]. Benitez et al. investigated the temperature-dependent diffusion coefficients of Li^+^ in the main SEI components of LIBs with silicon (μSi) anodes [79]. They found that at 250 K, the diffusion coefficients of Li^+^ in LiF, Li_2_O, and Li_2_CO_3_ were 6.83 × 10^–17^, 1.71 × 10^–18^, and 1.00 × 10^–16^ m^2^ s^−1^, respectively. At 400 K, the corresponding values increased to 1.07 × 10^–15^, 3.63 × 10^–16^, and 1.68 × 10^–14^ m^2^ s^−1^, indicating that the diffusion coefficient of Li^+^ decreases significantly with decreasing temperature. Recently, the SEI separation factor ($S_{{{\mathrm{SEI}}}}$) has been proposed to quantitatively describe charge (Li^+^/e^−^) transport and desolvation process on SEI (Fig. 4d) [80]. It is determined by four key thermodynamic parameters affecting LT electrochemical reactions of SEI, including electron work function ($\Phi_{{\mathrm{e}}}$), Li^+^ transfer barrier ($\Delta V_{{{\mathrm{Li}}}}$), surface energy ($E_{{\mathrm{s}}}$), and desolvation energy ($\Delta E_{{\mathrm{d}}}$). As shown in Fig. 4e, Kelvin probe force microscopy (KPFM) was employed to investigate the electronic work function of the SEI layer. A platinum-coated conductive tip was used to acquire the KPFM images. Subsequently, the contact potential difference (*V*_CPD_) between the probe tip and the SEI layer was measured (Fig. 4f, g). The electronic work function ($\Phi_{{\mathrm{e}}}$) of the SEI can then be calculated using the following equation:

3 $$ V_{{{\mathrm{CPD}}}} = \frac{{\Phi_{{{\mathrm{tip}}}} - \Phi_{{\mathrm{e}}} }}{e} $$

where $\Phi_{{{\mathrm{tip}}}}$ represents the intrinsic electronic work function of the platinum probe tip. The electron work functions of the SEI layer formed in carbonate-based electrolytes with the addition of vinyl carbonate (VC) and lithium difluorophosphate (LiDFP) are 3.62 and 4.87 eV, respectively. The $S_{{{\mathrm{SEI}}}}$ is calculated using the following equation:

4 $$ S_{{{\mathrm{SE}}I}} = (\Phi_{{\mathrm{e}}} /\Delta V_{{{\mathrm{Li}}}} ) \times (E_{{\mathrm{s}}} /\Delta E_{{\mathrm{d}}} ) $$

Among them, $\alpha_{1} = \Phi_{{\mathrm{e}}} /\Delta V_{{{\mathrm{Li}}}}$ is a parameter characterizing the charge (Li^+^/e^−^) separation and migration capability within the SEI layer. A higher $\alpha_{1}$ indicates that the SEI layer enables faster Li^+^ conduction compared to e^−^, thereby suppressing Li^+^/e^−^ combination and e^−^ tunneling effects, preventing solvent reduction decomposition and the formation of organic component-rich SEI layers. $\alpha_{2} = E_{{\mathrm{s}}} /\Delta E_{{\mathrm{d}}}$ reflects the SEI layer's barrier capability against material (solvent molecule) migration. A higher $\alpha_{1}$ represents stronger solvent repulsion ability, helping to inhibit solvent co-intercalation at the SEI surface. For example, the carbonate-based electrolyte with LiDFP (as an additive) forms an Li₃PO₄-enriched inorganic SEI with an *S*_SEI_ of 4.89 × 10^3^, ensuring that a LiNi_0.8_ Co_0.1_Mn_0.1_O_2_ (NCM811) ||Gr cell retains 90.6% of its capacity after 180 cycles at − 20 °C. In contrast, the carbonate electrolyte with VC (as an additive) forms an OVC-based SEI with a significantly lower S_SEI_ of 0.15 × 10^3^, resulting in a capacity retention (CR) of only 9.8%.

### Acceleration of Li Plating and Li Dendrite Growth

**Fig. 5:**
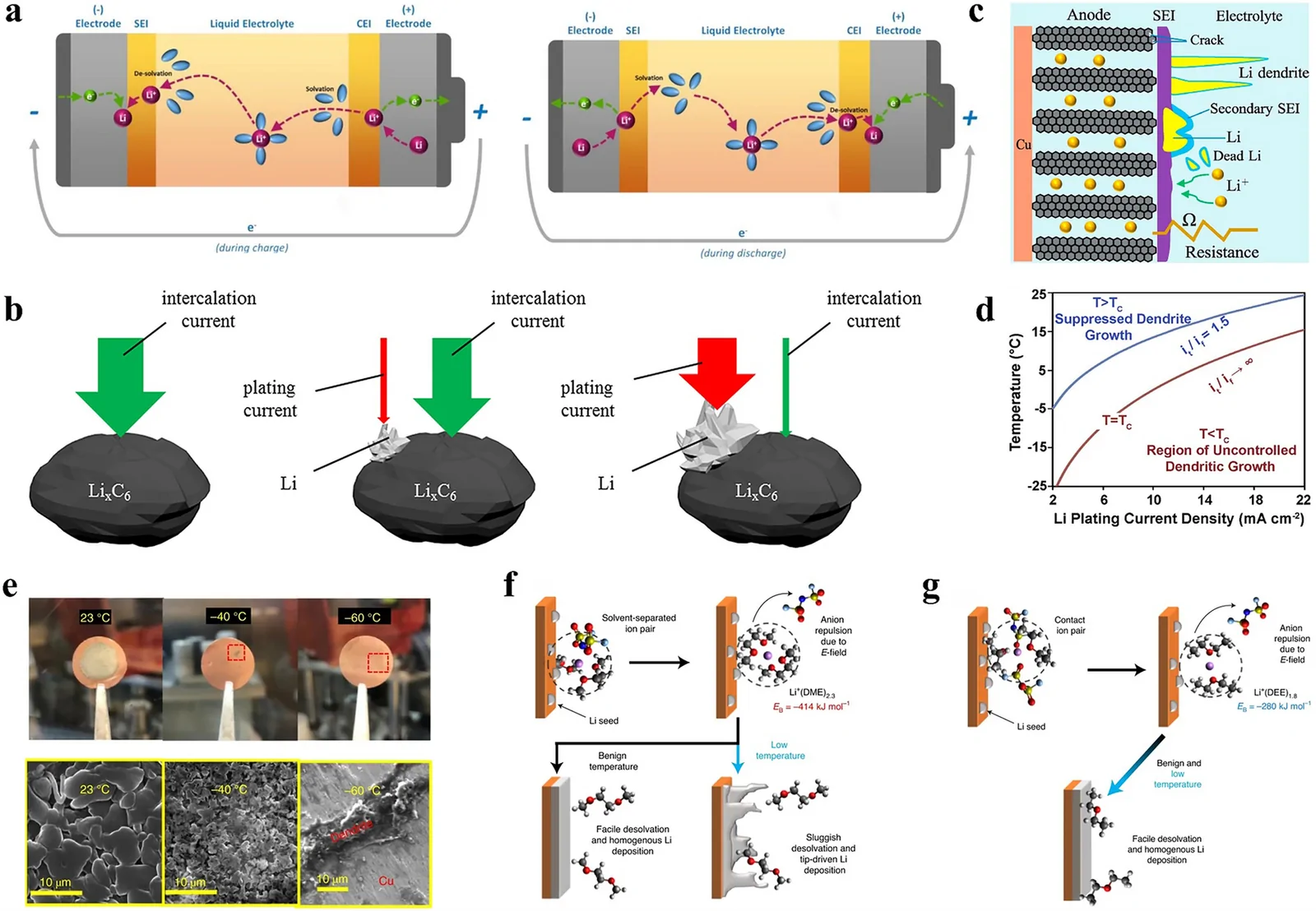
**a** Charge transfer process of Li⁺ during operation [81]**. b** A schematic representation of the competition between Li intercalation and Li plating reactions during charging [89]. **c** Degradation mechanism caused by Li plating on anode under low temperature [94]. **d** Variation of the dendrite growth rate ratio i_t_/i_f_ with temperature and Li plating current density [105]. **e** Optical image of the Cu current collector after deposition experiments in 1 M LiFSI DOL/DME electrolyte [41]. Schematic illustration of the desolvation mechanism and corresponding Li⁺/solvent binding energy in **f** 1 M LiFSI DOL/DME [41]. and **g** 1 M LiFSI DEE [41]

After completing transport through the SEI layer, Li⁺ ions move along specific diffusion pathways within the electrode material, as illustrated in Fig. 5a [81]. This phenomenon is referred to as the solid-state diffusion of Li ions. The solid-state diffusion kinetics of Li ions exhibit pronounced temperature dependence, as demonstrated by recent investigations [82–85]. Experimental evidence reveals a substantial reduction in Li ion diffusivity with decreasing temperature, following an Arrhenius-type relationship. Notably, the slow diffusion rate of Li⁺ on the anode side can lead to battery polarization and trigger Li plating [86]. In LIBs with Gr as the anode material, when the rate of Li⁺ reduction to metallic Li does not match the rate of Li⁺ intercalation into the Gr interlayers, some Li ions cannot be promptly intercalated and instead deposit as metallic Li on the Gr surface. This phenomenon is referred to as Li plating [87]. The fundamental cause of Li plating is the large overpotential induced by anode polarization [88, 89]. Due to the low lithiation potential of Gr (0.01 to 0.25 V vs. Li⁺/Li), the slow solid-phase diffusion at LTs leads to anode polarization, causing the electrode potential to shift further negatively until it falls below 0 V versus Li⁺/Li. Under these conditions, the Li plating reaction emerges as a competitive process to the intercalation reaction (Fig. 5b) [89]. The deposition of metallic Li becomes thermodynamically more favorable, thereby exacerbating the Li plating phenomenon. Battery polarization is generally classified into three categories [90, 91]: (1) Arises from the intrinsic resistance of various battery components, including electrode materials, electrolyte, separator, and interfacial contact resistance among different components. (2) Stems from the kinetic limitations of the electrochemical reaction itself and is associated with the activation energy of the reaction. (3) Occurs due to concentration gradients between the reactants near the electrode surface and those in the bulk solution, induced by the electrochemical reaction. Each of these polarization mechanisms contributes to the enhanced propensity for Li plating phenomena through distinct pathways. In the competition between the Li plating reaction and the intercalation reaction, factors such as slow desolvation processes and restricted Li⁺ transport within the SEI layer contribute to increased anode polarization. These factors enhance the competitive advantage of the lithium plating reaction, thereby exacerbating Li plating. Only by improving the solid-phase diffusion rate at the anode can the competitive advantage of the intercalation reaction be strengthened, thereby reducing the likelihood of l Li plating [92]. The study by Lüders et al. further demonstrated that LT conditions significantly heighten the risk of Li plating [93]. Utilizing static voltage measurements and neutron diffraction techniques, they investigated the Li plating characteristics of commercial 18,650 batteries at − 2 °C. The results revealed that Li deposition on the Gr surface became pronounced when the charging rate exceeded 0.5 C.

Li plating not only accelerates the degradation of battery performance but also introduces a series of severe safety risks (Fig. 5c) [94, 95]. First, once Li plating occurs, the exposed metallic Li continuously reacts with the electrolyte, forming additional SEI. This process not only depletes the electrolyte and reduces the amount of active Li but also increases the internal resistance of the battery, thereby accelerating performance degradation. Second, the non-uniform nucleation process and uneven current density distribution promote the dendritic growth of deposited metallic Li. These Li dendrites may further penetrate the separator, leading to internal short circuits and posing a severe threat to battery safety [96, 97]. A portion of the plated metallic Li cannot participate in the battery reactions during cycling and transforms into "dead Li," leading to irreversible capacity degradation [98–100]. Additionally, the side reactions between metallic Li and the electrolyte generate a significant amount of gas, increasing the internal pressure of the battery and thereby compromising the structural stability of the battery [97].

In LMBs, the fundamental electrode reactions at the LMA involve the plating and stripping of Li^+^, in contrast to the intercalation mechanism of conventional Gr anodes. For the LMA, Li plating is an inherent part of its normal operation. Therefore, during LT cycling, it does not experience the “Li plating” issue observed in Gr anodes. However, this does not imply that LMBs are free from Li plating-related issues at LTs. Under such conditions, the risk of Li dendrite growth increases significantly [101–104]. The ratio of the current density at the dendrite tip to that on a flat Li surface (*i*_t_/*i*_f_) has been proposed as a metric to quantify the growth rate of Li dendrites. The temperature at which *i*_t_/*i*_f_ approaches infinity is defined as the critical temperature (*T*_c_) for uncontrolled Li dendrite growth. If the ambient temperature falls below *T*_c_, Li dendrites will grow rapidly (Fig. 5d) [105]. In addition, the electrolyte system has a key influence on the morphology of Li deposition at low temperature. This was evidenced by the study conducted by Holoubek et al. [41], who discovered that the desolvation process of Li ions is hindered under LT conditions, resulting in a tip-driven mode of Li deposition. This phenomenon leads to the rapid growth of Li dendrites in a 1 M LiFSI 1,3-dioxolane (DOL)/ 1,2-dimethoxyethane (DME) electrolyte system at − 40 and − 60 °C (Fig. 5e, f). In contrast, the 1 M LiFSI / diethyl ether (DEE) electrolyte system demonstrates a more uniform Li deposition behavior under these extreme LT conditions (Fig. 5g).

## Strategies for Enhancing the LT Performance of LIBs

The solvation structure of Li⁺ determines its desolvation energy barrier, as well as the composition and thickness of the SEI [106, 107]. Therefore, the electrolyte plays a crucial role during the charge transfer process, particularly for batteries operating under LT conditions. An ideal LT electrolyte must exhibit high ionic conductivity and a low freezing point at the macroscopic level, while maintaining a low desolvation energy barrier of the Li⁺ solvation structure and forming a stable SEI/CEI at the microscopic level [108, 109]. In response, considerable research has focused on improving the LT performance of LIBs via the development of advanced electrolytes. The following sections summarize optimization strategies from the aspects of electrolyte regulation, interfacial engineering, and AI-assisted design.

### Li salts for LT Electrolytes

#### Conventional Li Salts

**Fig. 6:**
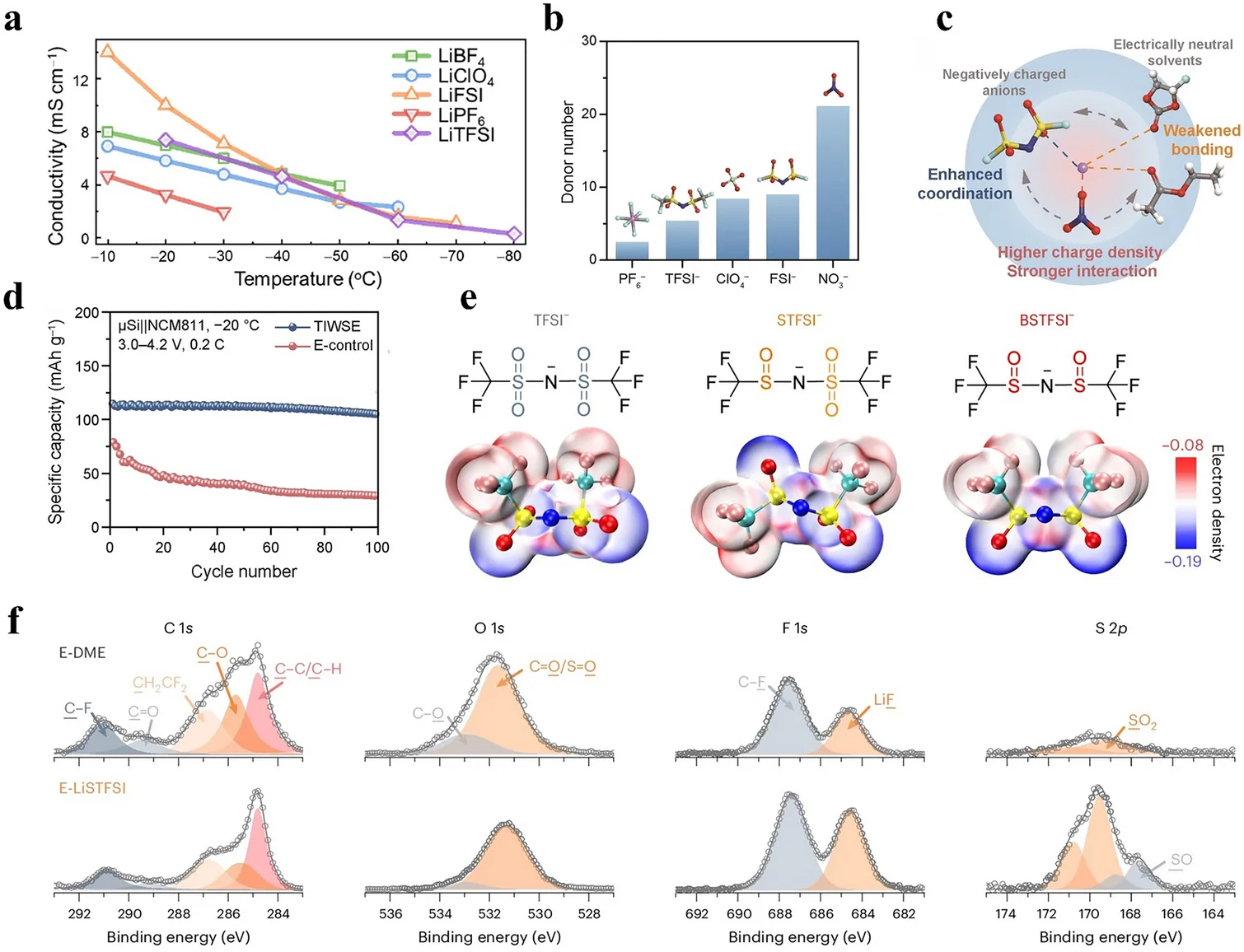
**a** Ionic conductivity of electrolytes employing different Li salts [45]. **b** Comparison of DN of various anions [131]. **c** Schematic diagram of the competitive coordination of Li^+^, anions and solvents regulated by NO_3_^–^ in TIWSE [131]. **d** Cycling performance μSi || NCM811 full cells at 0.2 C [131]. **e** Chemical structures comparison of the three anions [133]. **f** XPS analysis for the NMC811 cathodes cycled in different electrolytes for 30 cycles [133]

As a key component of LT electrolytes, Li salts determine the solvation structure, Li⁺ transport behavior, and interfacial stability, making their rational selection critical to electrolyte performance [110–114]. An ideal Li salt should possess characteristics such as low dissociation energy, high solubility, and excellent stability. The properties of commonly used Li salts are shown in Table 1. Although LiPF₆ exhibits poor thermal stability, high sensitivity to moisture, and lower conductivity compared to other Li salts (Fig. 6a), it is widely used in commercial electrolytes due to its excellent overall performance, including strong electrochemical stability and non-corrosiveness to Al current collectors [115–117]. Lithium tetrafluoroborate (LiBF₄) is primarily used as an additive due to its excellent high-temperature stability and outstanding LT performance. However, its poor compatibility with Gr anodes and low ionic conductivity limit its application as a standalone Li salt. Consequently, LiBF₄ is typically combined with Li salts that exhibit higher conductivity to enhance overall electrolyte performance [118]. Lithium bis (trifluoromethane sulphonyl) imide (LiTFSI) exhibits a high degree of dissociation due to the presence of electron-withdrawing groups (-CF₃SO₂) and a conjugated structure, which delocalizes the negative charge on the anion, thereby reducing the binding energy between the cation and anion [119, 120]. This reduced binding energy enables LiTFSI to dissociate effectively even in solvents with low DC, thereby increasing the concentration of free Li⁺ ions and enhancing ionic conductivity. However, when the voltage exceeds 3.7 V, LiTFSI tends to severely corrode the Al current collector, posing significant challenges for its widespread application [121]. LiFSI and LiTFSI share similar molecular structures and exhibit high solubility, strong dissociation ability, and excellent chemical stability. For example, LiFSI dissolved in acetone (DMK) with the addition of appropriate film-forming additives yielded a DMK-based electrolyte with an ionic conductivity exceeding 10 mS cm^−1^ at − 40 °C [122]. Notably, LiFSI demonstrates a higher corrosion potential for Al current collectors, reaching up to 4.2 V. As a result, LiFSI holds significant potential for improving the LT performance of electrolytes [123].

**Table 1:** Properties of Li salts used in LIBs electrolyte systems

Salt	Structure	Melting point (℃)	Molar mass	Dissociation energy (kJ mol^−1^)	Characteristics
LiPF_6_		200	151.9	439	Broad electrochemical stability window; non-corrosive toward Al current collectors; poor thermal stability; high sensitivity to moisture; SEI layer degradation caused by HF generated from side reactions
LiBF_4_		293–300	125.91	596	Low moisture sensitivity; excellent thermal stability; lower ionic conductivity compared to LiPF₆
LiBOB		> 300	193.79	/	Excellent thermal stability; superior film-forming capability; low solubility
LiDFOB		265–271	161.79	494	Excellent film-forming properties; superior LT performance; effective inhibition of electrolyte oxidation
LiTFSI		234–238	286.9	514	High solubility and ionic conductivity; low susceptibility to hydrolysis; corrosive behavior toward Al current collectors
LiFSI		124–128	187.1	344	High solubility; excellent LT performance; superior thermal stability; poor solvent compatibility; high cost

Lithium bis (oxalato) borate (LiBOB) is one of the most commonly used film-forming additives in LT electrolytes, as it promotes the formation of a dense SEI and serves as an HF scavenger, preventing HF-induced transition metal (TM) dissolution and maintaining the structural integrity of the electrode [124]. LiDFOB [125], a hybrid form of LiBOB and LiBF₄, can not only form a stable SEI on the anode but also generate a dense CEI on the cathode surface. Therefore, LiDFOB is widely used as an additive in LT electrolytes [126, 127]. For instance, when LiDFOB is added to conventional carbonate electrolytes, it facilitates the formation of borates on the cathode surface and promotes the generation of high concentrations of P–O species on the Gr surface, thereby enhancing the cycling performance of the battery [126]. In addition, LiDFOB has also been applied in ether-based electrolytes [128]. A concentration of 0.02 M LiDFOB was added to an electrolyte consisting of 3 M LiFSI dissolved in DME. This addition promotes the formation of an SEI rich in Li_3_N and LiF, as well as a dense CEI [129]. Yang et al. also demonstrated that the DFOB⁻ anion can actively participate in the primary solvation shell of Li⁺ and promote the formation of both the SEI and CEI [77].

Lithium nitrate (LiNO₃) is also widely used as an additive. The NO₃⁻ anion, with its high donor number (DN), preferentially occupies the inner Li⁺ solvation shell, effectively competing with the solvent for Li⁺ coordination. This promotes the formation of an inorganic-rich SEI and accelerates the Li⁺ desolvation kinetics [130]. Hu et al. [131] dissolved LiFSI and LiNO₃ in a mixed solvent of ethyl acetate (EA) and fluoroethylene carbonate (FEC) to prepare a temperature-inert weakly solvating electrolyte (TIWSE). As an additive, LiNO₃ introduced NO₃⁻ anions with high DN into the TIWSE, thereby promoting the formation of an anion-rich solvation structure (Fig. 6b, c). Benefiting from the incorporation of TIWSE, the micro-sized μSi || NCM811 full cell achieved a CR of 91.8% after 100 cycles at − 20 °C (Fig. 6d).

To enhance the LT performance of electrolytes, an effective strategy is to modify commonly used Li salts. For example, a 2 M electrolyte obtained by dissolving modified lithium cyano (trifluoromethanesulfonyl) imide (LiCTFSI), derived from LiTFSI, in a PC and FEC mixture (volume ratio of 7:3) not only enhances the high-voltage stability of the Al current collector but also enables the NCM811 || Gr battery to deliver 168 mAh g⁻^1^ at − 20 °C [132]. Yang and his collaborators synthesized an asymmetric lithium (trifluoromethanesulfinyl) (trifluoromethanesulfonyl) imide (LiSTFSI) (Fig. 6e). X-ray photoelectron spectroscopy (XPS) testing and time-of-flight secondary ion mass spectrometry (TOF–SIMS) analysis revealed that STFSI⁻ can promote the formation of a double-layer inorganic CEI derived from anions (Fig. 6f). The ether-based electrolyte containing LiSTFSI was applied in a NMC811|| Li full cell, enabling stable operation for over 2000 cycles at − 20 °C, with a CR of 85.7% [133].

#### Salt-Regulation Strategy

**Fig. 7:**
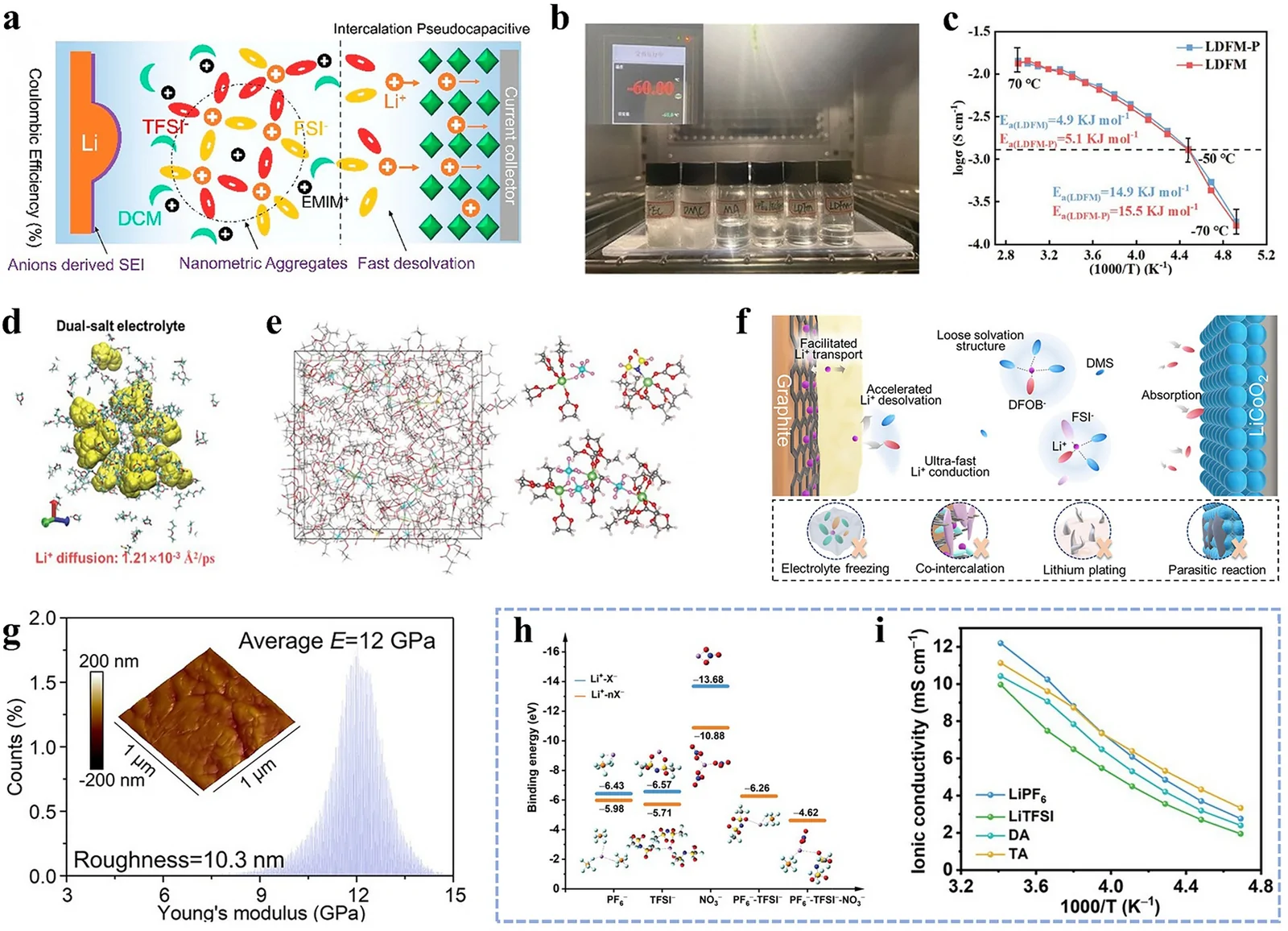
**a** Schematic illustration of the solvation structure and fast interfacial kinetics at the interface [140]**. b** Photographs of different solvents and electrolytes at − 60 °C [107]. **c** Arrhenius plot for ionic conductivity (σ) and activation energy (*E*_a_) for Li^+^ diffusion [107]. **d** Diffusion pathways and corresponding diffusion coefficients of Li^+^ for dual-salt electrolyte [141]. **e** Possible Li^+^ solvation structures of the dual- salt electrolyte obtained from MD simulation [141]. **f** Schematic illustration of the solvation structure in dual-salt electrolytes [142]. **g** Young’s modulus distribution and AFM Images of SEI at − 50 °C [142]. **h** Binding energies of Li^+^ with PF6^−^, TFSI^−^, NO_3_^−^, PF6^−^–TFSI^−^, and NO3^−^–PF6^−^–TFSI^−^ [143]. **i** Ionic conductivities of different electrolytes [143]

Recent studies have demonstrated that introducing multiple salts or mixed solvents to form a high-entropy solution can alter diverse solvation structures and regulate the interfacial formation process [134–136]. The mixing of multiple Li salts increases the disorder of the electrolyte system, thereby enhancing the solubility of Li salts at LTs. Meanwhile, anions are introduced into the solvation sheath of Li⁺ to form contact ion pairs (CIPs) and aggregates (AGGs), which suppress solvent decomposition and facilitate the formation of anion-derived interfacial films [137–139]. The anion-enriched solvation structure facilitates the desolvation process of Li⁺ at the electrolyte–electrode interface, thereby enhancing ionic transport capability under LT conditions (Fig. 7a) [140]. Dual-salt electrolytes leverage complementary properties of different Li salts, enabling synergistic optimization of solvation structure, ionic conductivity, and interfacial stability under LT conditions. For example, Chen et al. reported a dual-salt electrolyte system, in which lithium difluorophosphate (LiPO_2_F_2_) was added to a 1 M LiTFSI-dimethyl carbonate (DMC)/ FEC/ methyl acetate (MA) electrolyte solution [107]. Due to its lower solubility and stronger binding affinity with Li⁺, LiPO₂F₂ exhibited a pronounced tendency to integrate into the primary solvation shell of Li⁺. Moreover, the synergistic interaction between LiTFSI and LiPO₂F₂ established a dual-anion-driven mechanism, effectively weakening the interaction between Li⁺ and solvent molecules, thereby reducing the de-solvation energy barrier at LTs. This electrolyte remained unfrozen at − 60 °C and exhibited an ionic conductivity of 1.3 mS cm⁻^1^ at − 50 °C (Fig. 7b, c), enabling the stable cycling of NCM811 || Li cells at − 50 °C. Molecular dynamics (MD) simulations have shown that the addition of LiFSI to a LiPF6-based carbonate electrolyte facilitates the formation of a Li⁺ solvation structure modified with multiple anions and fewer solvent molecules. This optimized solvation structure effectively enhances the Li⁺ diffusion coefficient and reduces the Li⁺ desolvation energy (Fig. 7d, e). As a result, LiNi_0.52_Co_0.2_Mn_0.28_O₂ (NCM523) || Gr batteries can stably cycle 350 times at − 20 °C under a 4 C charging rate, maintaining a CR rate of 89% [141]. A dual-salt electrolyte, prepared by dissolving 0.6 M LiFSI and 0.4 M LiDFOB in dimethyl sulfite (DMS), which exhibits an extremely low melting point (− 141 °C) and a high DC (22.5), effectively combines the advantages of LiFSI and LiDFOB (Fig. 7f). Benefiting from the strong dissociation ability of LiFSI, its pairing with DMS facilitates rapid Li⁺ ion conduction at ultra-LTs. Meanwhile, the strong affinity between DFOB⁻ and Li⁺ ensures a fast desolvation process. The formation of a thin and inorganic-rich SEI can also be facilitated by such dual-salt electrolyte (Fig. 7g). Using this electrolyte, a 1 Ah LiCoO_2_ (LCO) || Gr battery delivers a reversible capacity of 0.86 Ah at − 50 °C and can even operate normally at temperatures as low as − 78 °C [142]. Ternary-salt electrolytes have garnered significant research attention due to their ability to enhance the LT performance of LIBs. Cheng and collaborators developed a ternary anion (TA) electrolyte by dissolving LiNO₃, LiPF₆, and LiTFSI in a mixed solvent of (THF) and FEC, which exhibits rapid charge transfer kinetics and low Li⁺ desolvation energy barriers. The strong interaction between NO₃⁻ and Li⁺ significantly diminishes the participation of weakly coordinated PF₆⁻ and TFSI⁻ anions in the Li⁺ solvation sheath, yielding a Li⁺-anion binding energy of − 4.62 eV (Fig. 7h). Simultaneously, the TA electrolyte demonstrates an ionic conductivity of 3.39 mS cm⁻^1^ at − 60 °C (Fig. 7i). Benefiting from these superior properties, the NCM811|| Li full cell retains a capacity of 103.85 mAh g⁻^1^ at − 60 °C [143].

Table 2 summarizes the electrochemical performance of various electrolytes developed through Li salt modulation strategies. These studies demonstrate that the type and concentration of Li salts have a significant influence on the regulation of Li⁺ solvation structures, which in turn determine the desolvation energy barrier and associated kinetic behavior. The selection of appropriate Li salts or the optimization of their concentration can modulate the formation of anion-dominated solvation structures, which helps to lower the desolvation energy barrier and thereby improve LT performance. Developing new Li salts with a high dissociation degree is one of the key directions for designing high-performance LT electrolytes. It is necessary to further investigate their mechanisms in regulating the Li⁺ solvation structure and reducing the desolvation energy barrier. Furthermore, multi-Li salt systems, by modulating the interactions of various anions, can enhance ion transport efficiency while optimizing the structure of the SEI/CEI. This enables superior electrochemical performance at LTs. However, the potential of multi-salt systems for optimizing LT performance remains largely unexplored. In-depth research into the interaction mechanisms between different Li salts is necessary. Future research needs to integrate experimental and theoretical computational approaches to conduct an in-depth analysis of the relationship between the solvation structure and LT electrochemical behavior, thereby guiding the precise design and optimization of Li salt systems.

**Table 2:** Electrochemical performance of various electrolyte formulations regulated by Li salt tuning strategies

Electrolyte formulation	Cell type	CR at LT (current density)	Cycle number	Refs
0.9 M LiFSI + 0.1 M LiNO_3_ − EA/FEC (9:1 by vol)	NCM811 || μSi	91.8% at − 20 ℃ (0.2 C)	100	[131]
2 M LiCTFSI − PC/FEC (7:3 by vol)	NCM811 || Gr	92.1% at − 20 ℃ (0.1 C)	200	[132]
1 M LiFSI + 0.2 M LiNO_3_ + 0.3 M LiSTFSI–DME	NMC811 || Li	85.7% at − 20 ℃ (0.3 C)	2000	[133]
1 M LiFSI − DMC/FEC/MA (4: 3: 3 by vol) + 1 wt % LiPO_2_F_2_	NCM811 || Gr	72% at − 40 ℃ (0.2 C)	30	[107]
1.25 M LiPF_6_ − EC/PC/DEC/EP (2:1:2:5 by vol) + 2% VC + 0.5% DTD + 2% PS + 0.5% LiFSI	NCM523 || Gr	89% at − 20 ℃ (4 C)	350	[141]
0.4 M LiDFOB + 0.6 M LiFSI–DMS	LCO || Gr	90% at − 20 ℃ (2 C)	850	[142]
0.5 M LiPF_6_ + 0.5 M LiTFSI + 0.1 M LiNO_3_–THF/FEC (9:1 by vol)	NMC811 || Li	84.64% at − 30 ℃ (0.05 C)	50	[143]

### Solvent

#### Ester

**Fig. 8:**
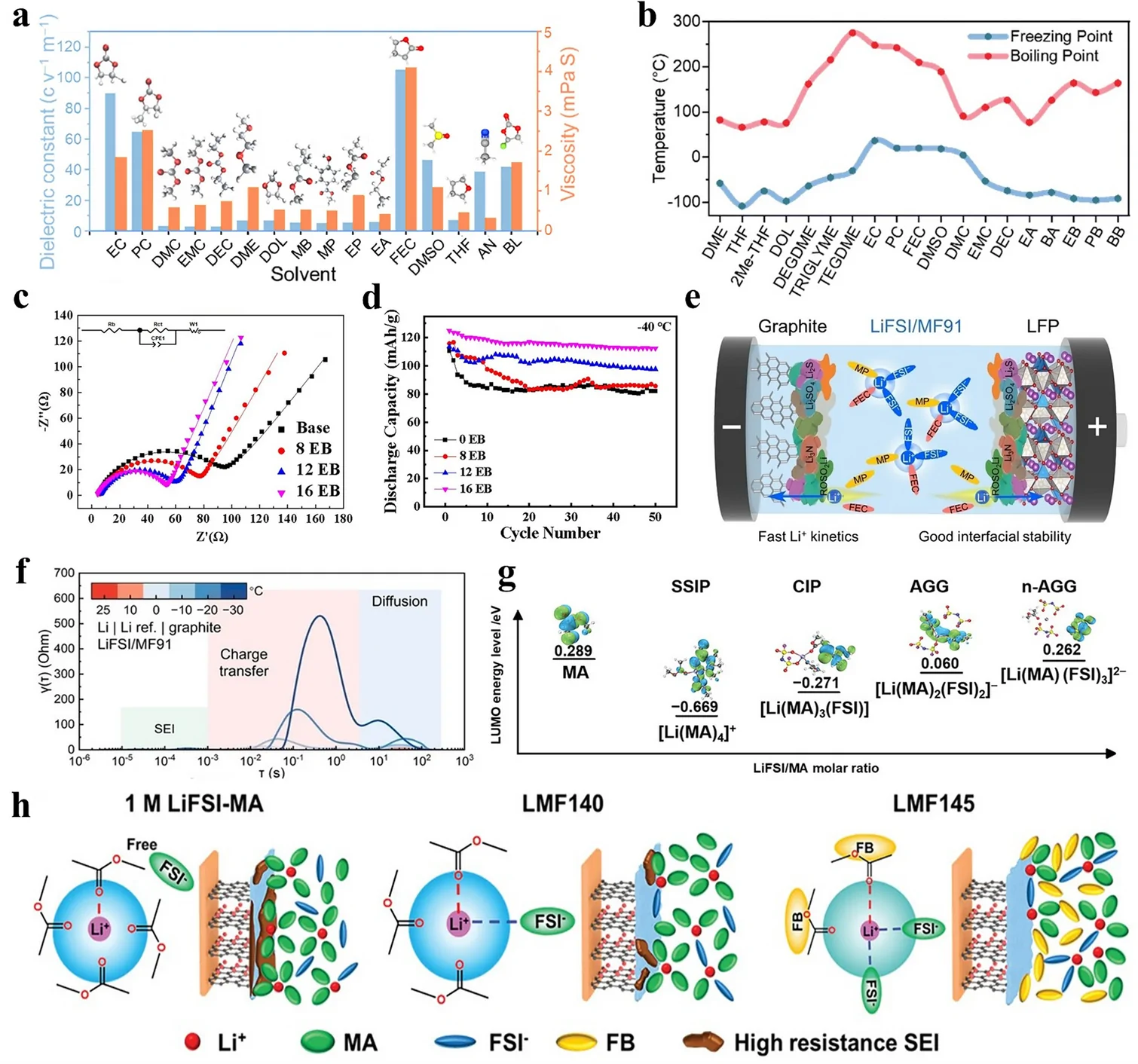
**a** Comparison of DC and viscosity of common solvents [45]. **b** Freezing and boiling points of common solvents [46]. **c** EIS impedance spectra for cells containing various electrolytes [157]. **d** Cycling profiles of electrolytes with varying EB content at − 40 °C [157]. **e** Schematic illustration of interfacial Li⁺ migration in the LFP || Gr cell [159]. **f** DRT curves of the Gr anode at different temperatures [159]. **g** Visual LUMOs and energy level of different solvation structures [164]. **h** Schematic illustration of solvation structures and interfacial models for different electrolytes [164]

Ester-based solvents are widely used in LT electrolyte systems due to their excellent overall performance [144, 145]. However, single-component ester solvents often suffer from high viscosity and insufficient interfacial stability, limiting their application under extremely LT conditions. Therefore, to expand the liquid-phase range of electrolytes and enhance their LT ionic conductivity, it is necessary to develop co-solvents with low melting points and low viscosity [146]. A variety of low-freezing-point carbonates, carboxylates, and fluorinated esters have been proposed to expand the LT liquid-phase range of electrolytes [85, 147, 148]. The freezing points, DC, and other properties of different solvents are shown in Fig. 8a and b.

Carbonates ester were the first organic solvents used in LIBs. Currently, commercial carbonate solvents can be classified into cyclic carbonates and linear carbonates. EC and PC are common cyclic carbonates. Previous studies have shown that the poor performance of commercial LIBs at LTs is primarily attributed to the commonly used EC solvent in electrolytes. Due to its high freezing point, EC significantly increases electrolyte viscosity below − 20 °C, thereby hindering ion transport [149]. Moreover, EC-based electrolytes exhibit strong interactions between the solvent and Li⁺, making the desolvation process more challenging [117, 150]. More critically, at LTs, the sharp decline in electrolyte conductivity and increased polarization can easily lead to Li metal deposition, resulting in continuous electrolyte consumption and even potential safety hazards [151]. Although EC has long been widely used due to its ability to form a stable SEI on Gr anodes, recent studies have shown that EC-free electrolyte systems can also exhibit excellent LT performance [51, 152]. DMC and diethyl carbonate (DEC) have lower viscosity, which facilitates the migration of Li⁺ ions at LTs, thereby enhancing the battery's LT performance. However, their limited ability to dissolve Li salts and insufficient wettability with electrode materials can compromise the stability of the SEI to some extent [71]. The performance of EMC falls between that of EC and DMC/DEC, exhibiting balanced properties in terms of Li salt solubility, viscosity, and boiling points [61]. EC is often mixed with linear carbonates such as DMC and DEC to achieve better LT performance. For example, Xu et al. investigated the effect of electrolyte composition on the performance of LIBs under different environmental temperatures [153]. The results indicated a subtle relationship between LT performance, the critical temperature of − 20 °C, and the EMC content. Specifically, when the temperature drops below − 20 °C, further increasing the EMC content in the co-solvent system is necessary to enhance ionic conductivity and CR. Ultimately, a ternary co-solvent system with 1.0 M LiPF₆ in EC/PC/EMC (1:1:8) was found to provide the broadest applicable temperature range. Although co-solvent systems composed of linear and cyclic carbonates are widely used in commercial electrolytes, their high flammability and the instability of linear carbonates with LMA limit their application in LT LIBs [154, 155]. Therefore, it is necessary to develop strategies to enhance the thermal stability of linear carbonates, such as incorporating flame retardants.

Compared to carbonates, carboxylate esters exhibit lower melting points, lower viscosity, and higher DC. Typical linear carboxylate esters, such as ethyl butyrate (EB), methyl acetate (MA), ethyl acetate (EA), methyl propionate (MP), and ethyl propionate (EP), have been proven to enhance the LT performance of LIBs to some extent [20, 156]. For instance, the addition of EB significantly lowered the Li^+^ transport resistance under LT conditions, thereby enhancing the cycling stability [157] (Fig. 8c, d). An EA-based electrolyte has been proposed to passivate deposited Li metal at LTs, enabling stable cycling at − 20 °C [158]. However, its strong crystallization tendency below − 40 °C severely limits the operation of LIBs in ultra-LT environments. MP is used as the primary solvent due to its low freezing point, moderate boiling point, and wide electrochemical stability window [20]. Yan et al. reported a novel ester-based electrolyte, in which LiFSI is dissolved in a mixed solvent of MP and FEC with a volume ratio of 9:1 (Fig. 8e) [159]. This electrolyte exhibits high ionic conductivity and fast interfacial kinetics at LTs. The distribution of relaxation time (DRT) shows that the electrolyte exhibits faster Li⁺ transfer at LTs (Fig. 8f). Thanks to this advantage, the LFP || Gr pouch cell operates stably even at − 80 °C. As a short chain ester, MA possesses low viscosity (0.37 cP), a low freezing point (− 98 °C), and excellent oxidative stability (> 5 V vs. Li/Li⁺) [51]. Scanning electrochemical microscopy (SECM) and atomic force microscopy (AFM) analyses show that MA, when used as an additive, can enhance the mechanical properties of the SEI [160]. In addition, MA-based electrolytes exhibit exceptionally high ionic conductivity (25 mS cm⁻^1^ at room temperature), making them highly promising LT solvents [161]. However, their poor interfacial stability often results in shortened cycle life, posing significant challenges for practical application. The electrochemical stability of MA-based solvents can be improved by controlling the amount of easily reducible MA molecules or by introducing film-forming additives [162]. For instance, the incorporation of MA as a co-solvent in carbonate-based electrolytes has been shown to enhance ionic conductivity at LTs while maintaining full-cell stability [163]. Dahn et al. demonstrated that MA-based electrolytes with film-forming additives significantly improve the rate capability and cycling stability of LIBs under LT conditions [161]. Recently, Lei et al. reported a strategy to enhance the reductive stability of MA-based electrolytes by leveraging dipole–dipole interactions between solvents [164]. They found that the lowest unoccupied molecular orbital (LUMO) energy level of the coordinated MA molecules exhibits a “V”-shaped trend with increasing anion coordination. Upon the incorporation of anions into the primary solvation shell of Li⁺, the LUMO level increases significantly, indicating an enhancement in the reductive stability of the electrolyte (Fig. 8g). Accordingly, they formulated an electrolyte with a molar ratio of LiFSI: MA: fluorobenzene (FB) as 1:4:5, referred to as LMF145. The nonpolar FB extracts the solvated MA molecules from the solvation shell, thereby promoting the formation of anion-dominated solvation structures to enhance the reductive stability of the electrolyte (Fig. 8h). Using this electrolyte, LCO || Gr cells achieved a 90% CR after 1,000 cycles at − 20 °C and 1 C, and maintained 91% of their room-temperature capacity when discharged at 0.05C under ultra-LTs of − 60 °C. In summary, the design of LT electrolytes requires precise control over the types and ratios of carboxylates to achieve an optimal balance between ionic conductivity and interfacial stability, thereby enhancing the discharge capability and cycling life of the battery.

**Fig. 9:**
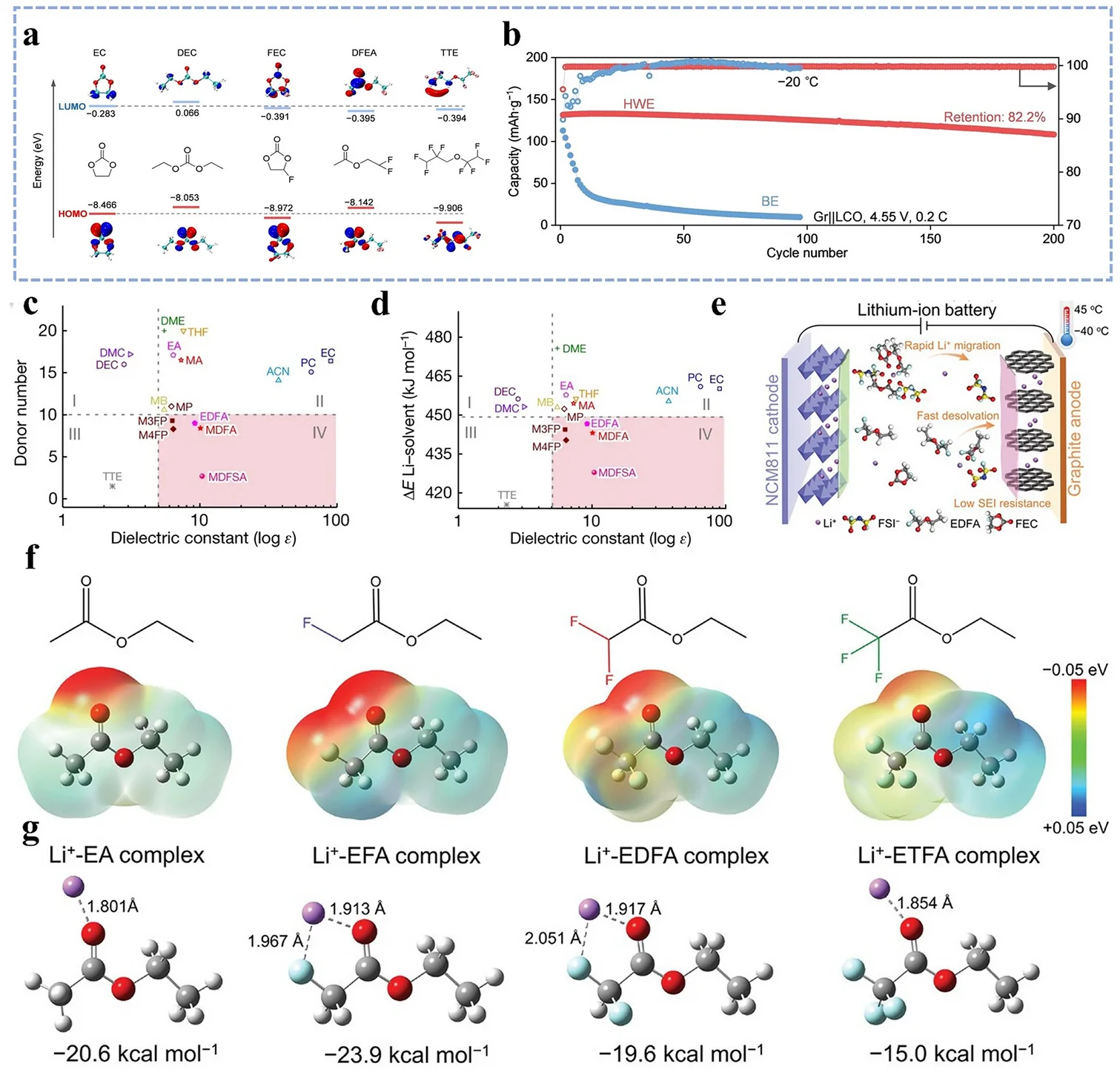
**a** Chemical structures and the corresponding molecular orbital energies of various solvents [148]. **b** Cycling performance of LCO || Gr full-cells at − 20 °C [148]. **c** Relationship between DN and DC of solvent [108]. **d** Relationship between Li^+^–solvent binding energy and DC [108]. **e** Schematic illustration of an NCM811 || Gr cell containing EDFA-based electrolyte [144]. **f** Electrostatic potential (ESP) maps of EA and fluorinated EA [144]. **g** Coordination structures and binding energies of Li^+^–solvent complexes [144]

Studies have shown that incorporating fluorinated groups into esters can effectively enhance the LT performance of LIBs [20, 165]. This improvement is attributed to the ability of fluorinated groups to lower the highest occupied molecular orbitals (HOMOs) of the solvents, thereby ensuring high oxidative stability. Additionally, they promote the formation of LiF-rich interphases [166–168]. Notably, fluorine atoms possess strong electron-withdrawing capability and low polarizability, which endows fluorinated solvents with weak solvation ability, effectively reducing the desolvation energy of Li⁺ [169]. For example, FEC exhibits a relatively low HOMO energy level (Fig. 9a), endowing FEC-based electrolytes with enhanced oxidative stability. When applied in LCO || Gr full cells, they demonstrate high cycling stability at − 20 °C (Fig. 9b) [148]. Fluorine atoms with high electronegativity can be covalently introduced into low-freezing-point carboxylate molecules to achieve a balance between a wide liquid-phase temperature range and weak affinity for Li⁺. For example, ETFA has been shown to enable rapid desolvation at LTs [170]. The EMC: 2,2,2-trifluoroethyl acetate (TFA) (30:70 vol%)-based electrolyte exhibits superior ionic conductivity at − 30 °C compared to the traditional EC: EMC (30:70 vol%) electrolyte [171]. Studies have shown a strong correlation between the DN and the Li⁺–solvent binding energy, with solvents exhibiting higher DN values typically demonstrating stronger Li⁺–solvent interactions [172] (Fig. 9c, d). Wang et al. leveraged this characteristic to screen fluorinated esters that possess moderate Li⁺–solvent binding energies and suitable salt dissociation capabilities. Among them, methyl difluoroacetate (MDFA) and methyl 2,2-difluoro-2(fluorosulfonyl)acetate (MDFSA) were identified as promising primary solvents for formulating LT electrolytes. The results demonstrated that NMC811||Gr cells using these electrolytes achieved an average coulombic efficiency (CE) exceeding 99.9% during cycling at − 30 °C [108].

Although fluorination strategies can effectively weaken the interaction between Li⁺ and solvent molecules, the resulting weaker solvation affinity often compromises Li salt solubility and Li^+^ transport capability [173, 174]. Excessive fluorination can suppress the dissociation of Li salts and reduce ionic conductivity. Therefore, it is essential to achieve a balance between weak Li⁺ solvation and good ionic conductivity by regulating the degree of fluorination. Electrolytes formulated with fluorinated EA solvents featuring different degrees and positions of fluorination can yield distinct Li⁺ solvation structures. For instance, compared with mono-fluoro (–CFH₂), trifluoro (–CF₃), and methyl-side difluoro (–CF₂H) substituents, the ethoxy-side difluoro group (–OCH₂CF₂H) exerts a stronger electron-withdrawing effect. This facilitates the formation of a moderately coordinated solvation structure, accelerates Li⁺ desolvation at LTs, and induces the formation of a LiF-rich interphase, thereby significantly enhancing the LT performance of LIBs [175]. Xia et al. discovered that the moderately-fluorinated ethyl difluoroacetate (EDFA) exhibits lower Li⁺ binding energy compared to the less-fluorinated ethyl fluoroacetate (EFA), while outperforming the highly-fluorinated ETFA in Li salt dissociation capability (Fig. 9e) [144]. This achieves an optimal balance between weak solvation affinity and high ionic conductivity (Fig. 9f, g). The electrolyte based on EDFA exhibits a unique solvation sheath structure, enabling a 1.2 Ah NCM811||Gr pouch cell to deliver a discharge capacity of 790 mAh at − 40 °C. Linear sweep voltammetry (LSV) analysis revealed that, in contrast to the EA-based electrolyte with an oxidation potential of 4.6 V (vs. Li/Li⁺), electrolytes based on difluoro 2,2-difluoroethyl acetate (DFEA) and 2,2,2-trifluoroethyl acetate (TFEA) exhibit excellent oxidative stability, maintaining stability even at voltages as high as 5.5 V (Fig. 9f). Compared to TFEA, DFEA exhibits weaker anion–solvent interactions, which lowers the kinetic barrier for anion desolvation and suppresses co-intercalation of solvents into the Gr anode [176].

#### Ether

Ether-based electrolytes are considered one of the most promising candidates for LIBs due to their remarkable advantages under LT conditions [177, 178]. These advantages include excellent compatibility with LMA [179, 180], superior Li⁺ transport kinetics, extremely low viscosity, and ultra-low freezing points [181]. For example, THF, with its exceptionally low freezing point (− 108.4 °C) and high DN, ensures a high degree of Li salt dissociation and facilitates the formation of a stable interface on LMA, making it an excellent solvent for LT LMBs [182]. Despite the numerous advantages of ether-based solvents, their poor oxidative stability (< 4.0 V) poses a significant challenge when pairing with high voltage cathodes [183, 184]. Specifically, free ether molecules accumulated in the electric double layer can undergo sequential decomposition under the catalytic influence of high-valence Ni^4^⁺ ions present in Ni-rich cathode materials [185, 186]. Furthermore, ether-based electrolytes struggle to form an oxidation-resistant CEI at high voltages, which triggers interfacial degradation, TM dissolution, and capacity fading issues [187, 188]. DOL, as a cyclic ether solvent, exhibits significantly lower freezing point (− 95 °C) and viscosity (0.6 cP), thereby substantially enhancing reaction kinetics under LT conditions and demonstrating considerable application potential [41]. However, at LTs, DOL tends to polymerize with inorganic salts (e.g., LiBF₄, LiDFOB, and LiPF₆), leading to reduced ionic conductivity and deteriorated electrochemical performance [189, 190]. The addition of LiNO₃ to the electrolyte can suppress the polymerization of DOL through coordination between NO₃⁻ anions and the epoxy groups in DOL. However, this strategy compromises the ion transport rate at the electrode/electrolyte interface, as the strong interaction between NO₃⁻ and the epoxy groups increases the charge transfer resistance [191]. An electrophilic reagent, trimethylsilyl isocyanate (Si-NCO), was introduced into the DOL-based electrolyte as a water scavenger. It removes trace water through a nucleophilic addition reaction, thereby fundamentally protecting DOL from ring-opening polymerization. The DOL-based electrolyte containing Si-NCO enables LCO ||Li cells to retain 80% of their capacity after 150 cycles at − 40 °C [192]. In recent years, researchers have explored various strategies to enhance the oxidative stability of ether-based electrolytes. These include HCEs [37], LHCEs [193, 194], and WSEs [167, 195]. These strategies effectively reduce the coordination between Li⁺ and solvent molecules, allowing more anions to participate in the Li⁺ solvation structure and promoting the formation of CIPs and AGGs. Such solvation characteristics facilitate rapid Li⁺ desolvation and the formation of a stable, inorganic-rich interphase, thereby enhancing the LT performance of LIBs.

**Fig. 10:**
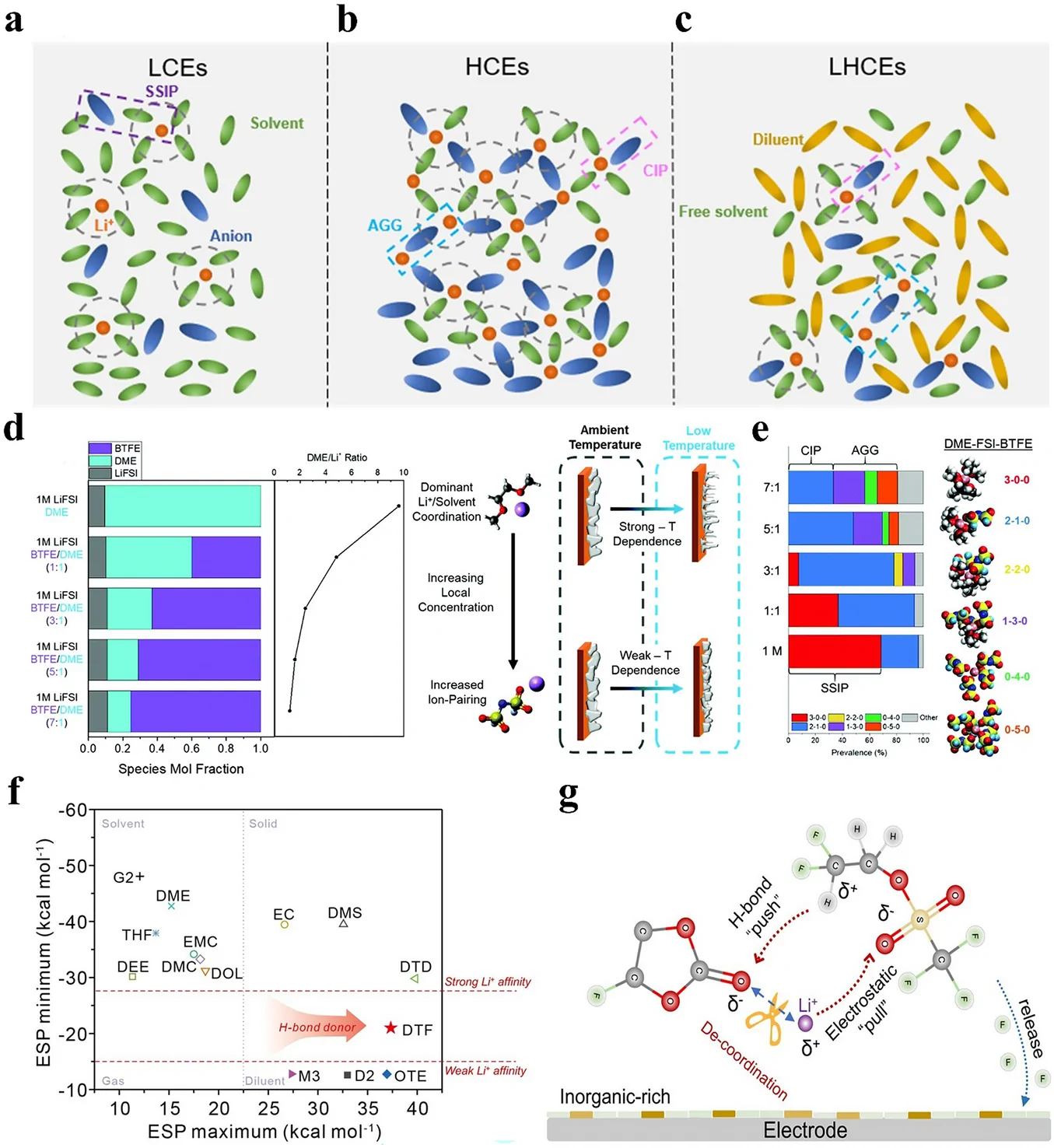
Li^+^ solvation structure of **a** LCEs, **b** HCEs, and **c** LHCEs [207]. **d** Schematic illustration of LHCEs with different localized concentrations [204]. **e** Solvation structure distribution and corresponding MD snapshots in various electrolytes [204]. **f** Electrolytes are selected based on the maximum and minimum values of the ESP surface [206]. **g** Schematic illustration of the “push–pull” mechanism of the DTF co-solvent [206]

In HCEs, most solvent molecules coordinate with Li ions, significantly reducing the number of free solvent molecules and thereby effectively suppressing undesirable side reactions of the solvent [196]. Moreover, in conventional low concentration electrolytes (LCEs), the solvation structure of Li ions scarcely involves anions and primarily exists in the form of solvent-separated ion pairs (SSIPs) (Fig. 10a). In contrast, due to the higher salt-to-solvent ratio in HCEs, anions are incorporated into the primary solvation shell of Li ions, forming CIPs and AGGs (Fig. 10b). This solvation structure not only lowers the desolvation energy barrier of Li⁺ but also facilitates the formation of a stable, inorganic-rich SEI [197, 198]. For example, a HCE formulated by dissolving 2 M LiTFSI and 2 M LiDFOB in DME can raise the cutoff voltage of LMBs to 4.3 V [199]. However, the dramatic increase in viscosity of HCEs can impede their electrochemical performance at LTs. Additionally, the high salt concentration leads to increased costs, which limits the practical application of HCEs.

To address the challenges faced by HCEs, non-solvating diluents have been introduced to construct LHCEs [187, 200]. These diluents do not disrupt the original coordination environment between Li⁺ and solvent molecules, thereby preserving the anion-involved solvation structures. As a result, the solvation characteristics of CIPs and AGGs can be maintained even at moderate salt concentrations (Fig. 10c). Diluent solvents, such as bis(2,2,2-trifluoroethyl) ether (BTFE), 1,1,2,2-tetrafluoroethyl-2,2,2-trifluoroethyl ether, and trifluorotriethyl ether (TTE), have been widely employed in LHCEs as inert diluents [201, 202]. For example, a LHCE composed of 1.3 M LiTFSI and 1.3 M LiFSI in DOL, diluted with TTE, enables LMBs to operate at voltages up to 4.6 V [203]. For LHCEs, the solvation structure is influenced by the local concentration. Chen et al. prepared a series of LT LHCEs with varying local concentrations by dissolving LiFSI in a mixed solvent of DME and BTFE, with the local concentration tuned by adjusting the volume ratio of BTFE to DME (Fig. 10d) [204]. In 1 M LiFSI / DME electrolyte, the solvation structure is predominantly composed of (SSIPs), along with a certain proportion of CIPs. In the SSIP configuration, the solvation shell of Li⁺ is entirely composed of solvent molecules, resulting in a high desolvation energy barrier and consequently poor LT performance. At moderate local concentrations (1: 1 and 3: 1 BTFE/DME ratio), the solvation structure primarily shifts to a CIP configuration. Notably, when the local concentration exceeds 4 M, a significant transformation in the solvation structure occurs. At higher local concentrations (7: 1 BTFE/DME ratio), AGGs become dominant (Fig. 10e). This effectively reduces the desolvation resistance, thereby enhancing the LT performance.

Tetrafluoro-1-(2,2,2-trifluoroethoxy) ethane (D2), serving as a diluent, can effectively modulate the Li⁺–solvent interactions. When incorporated into a fluorinated electrolyte system to form LHCEs, it enables a NCA || Li cell to achieve a discharge capacity of 96 m Ah g⁻^1^ at − 85 °C [123]. Moreover, studies have shown that the amphiphilic 1,1,2,2-tetrafluoro-3-methoxypropane (TFMP) can effectively accelerate the desolvation of Li⁺ at − 40 °C, thereby enhancing the kinetic performance [205]. Ren et al. selected 2,2-difluoroethyl trifluoromethanesulfonate (DTF) as the optimal co-solvent based on molecular ESP. Compared to hydrofluoroether diluents, DTF has a moderate ESP minimum value, which exhibits an appropriate affinity for Li^+^ (Fig. 10f). Furthermore, the sulfonate group of DTF can pull Li⁺ away from the solvent, while its difluoromethyl group, with a higher ESP maximum value than conventional diluents, can push solvent molecules away through competitive hydrogen bonding (Fig. 10g). This push–pull mechanism weakened the solvent coordination ability, thus facilitating the Li⁺ desolvation process. The electrolyte prepared by dissolving 1 M LiFSI in a mixed solvent of EMC/FEC/DTF at a volume ratio of 1.5:1.5:7 not only demonstrated a faster Li⁺ desolvation process at LTs but also forms a stable, inorganic-rich interface. Thanks to this property, the NMC811|| Li cells exhibited a 93% CR after 100 cycles at − 40 °C and 4.8 V [206].

**Fig. 11:**
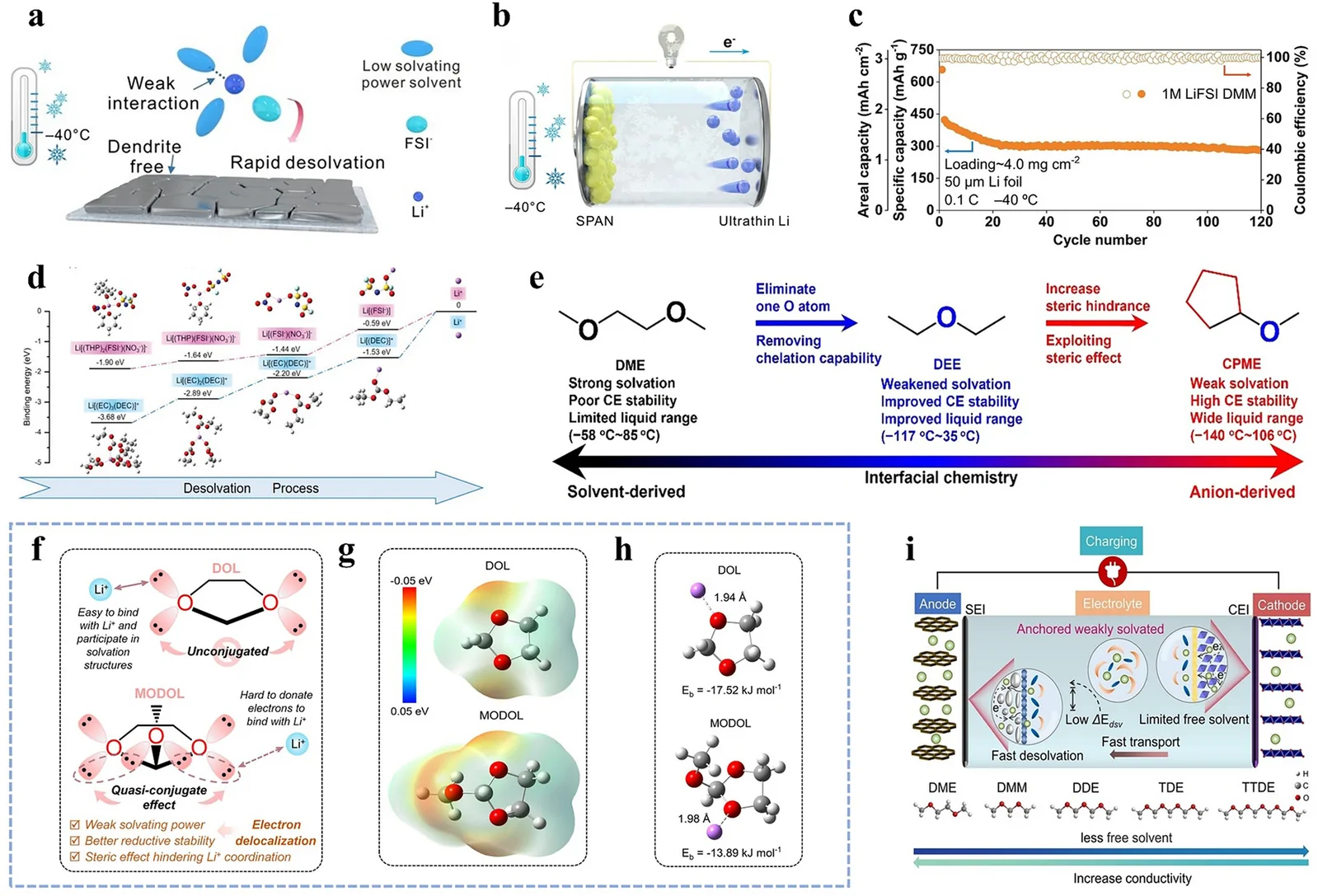
**a** Schematic illustration of the relationship between Li deposition morphology and solvent solvation ability at LTs [211]. **b** Schematic illustration of the SPAN || Li full cell and **c** its cycling performance at − 40 °C [211]. **d** Calculation of desolvation energy in different electrolytes [215]. **e** Schematic diagram of molecular design of the CPME solvent [217]. **f** Schematic diagram of molecular design of the MODOL solvent [218]. **g** ESP map of the DOL and MODOL solvent [218]. **h** Optimized coordination structures and binding energies of Li⁺–DOL and Li⁺–MODOL complexes [218]. **i** Schematic illustration of the mechanism of AWSEs [219]

Although the LHCE strategy has made considerable progress in enhancing the LT performance of LMBs, the fluorinated ether diluents commonly used in LHCEs are costly, have high densities, involve complex synthesis processes, and pose potential environmental concerns, which hinder their practical application in batteries [208, 209]. Ether-based WSEs are regarded as a cost-effective and practical strategy to enhance the LT performance of LMBs [210, 211], owing to the following advantages: (1) they do not require high salt concentrations and exhibit weak affinity toward Li⁺; (2) they are prepared via mature, low-cost processes; and (3) they can regulate the solvation structure to reduce the desolvation energy barrier of Li⁺. The solvents used in WSEs possess weak dissociation capabilities toward Li salts, exhibiting low solvating power. This leads to partial separation of cations and anions and weaker interactions with Li⁺, thereby allowing more anions to coordinate with Li⁺ and promoting the formation of a large number of CIPs and AGGs [212]. Meanwhile, this facilitates the formation of anion-derived SEI and CEI, thereby enhancing interfacial stability and improving LT performance of the electrolyte [213]. Notably, this also implies that even at relatively low salt concentrations (1 M), WSEs can effectively shift the interfacial chemistry from solvent-dominated to anion-dominated. The desolvation energy of Li^+^ primarily depends on the coordination strength of solvent molecules and their coordination number within the Li⁺ solvation shell. In electrolytes with low solvating power, the solvation shell of Li⁺ contains a higher proportion of anions, which facilitates a more rapid desolvation process [181, 214]. In contrast, strong solvent–Li⁺ interactions result in higher desolvation energy barriers, thereby impeding Li⁺ desolvation at LTs. Moreover, solvents with weak solvating abilities tend to promote the formation of uniform and compact Li deposition, as compared to those with strong solvating abilities. For example, electrolytes based on dimethoxymethane (DMM), which exhibit weak solvation ability, show lower desolvation energy, leading to uniform lithium deposition morphology and higher plating/stripping efficiency (Fig. 11a) [211]. In a WSE formulated by dissolving 1 M LiFSI in DMM, the coordination between Li⁺ and anions becomes more pronounced, resulting in lower desolvation energy for the DMM-based electrolyte. Owing to this advantage, the sulfurized polyacrylonitrile (SPAN) full cell employing this electrolyte exhibited favorable cycling performance at − 40 °C (Fig. 11b, c). Traditional ether solvents coordinate with Li⁺ primarily through the lone pair electrons on the ether oxygen atoms, forming various types of coordination structures. Common coordination modes include monodentate and multidentate coordination. Linear ethers exhibit strong interactions with Li⁺ due to their high electron density on oxygen atoms and multidentate chelation effects, resulting in solvent-dominated solvation structures. In contrast, cyclic ethers theoretically possess weaker coordination with Li⁺ due to their monodentate coordination and lower electron density on oxygen atoms. However, WSEs based on such monodentate cyclic ethers generally exhibit lower ionic conductivity and poor oxidative stability at LTs. To this end, Li and his collaborators developed a WSE suitable for LT LMBs, using LiFSI as the primary Li salt, tetrahydropyran (THP) as the main solvent, and FEC and LiNO₃ as additives. As a monodentate cyclic ether, THP exhibits weak affinity toward Li⁺. Under the synergistic effect of FEC and LiNO_3_, the electrolyte demonstrated a low desolvation energy barrier (Fig. 11d) and a high ionic conductivity of up to 0.73 mS cm⁻^1^ at an ultra-low temperature of − 50 °C. When applied in full cells based on NMC811 || Li, the electrolyte enabled a CR of 87% after 100 cycles at − 40 °C and 4.5 V [215].

**Fig. 12:**
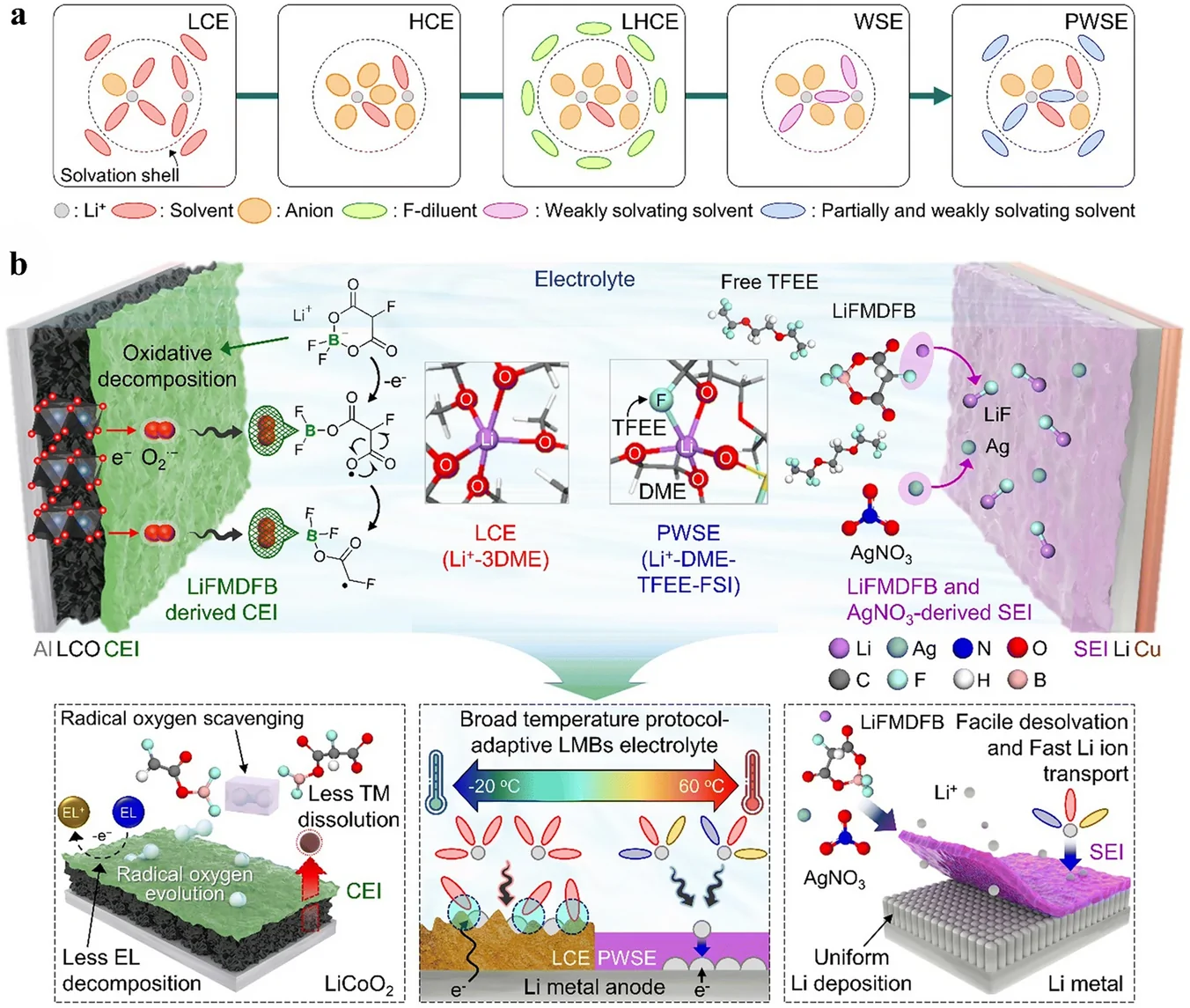
**a** Schematic illustration of the solvation structure of PWSE [114]. **b** Schematic illustration of the mechanism by which LiFMDFB and AgNO_3_ additives are incorporated into PWSE for LCO||Li full cells [114]

Rational molecular design to regulate the solvation ability of solvents is considered a feasible strategy, which includes reducing the number of oxygen atoms, shortening the alkyl chain of the main backbone, and increasing steric hindrance [211, 216]. For example, CPME, obtained by eliminating an oxygen atom and introducing steric hindrance, exhibits a relatively weak solvation ability (Fig. 11e). In CPME-based WSEs, CIPs and AGGs dominate the solvation structure, facilitating the formation of anion-derived, inorganic-rich SEI. At − 20 °C, LFP || Li cells employing this electrolyte display stable cycling performance [217]. Dong et al. reported a fluorine-free molecular design strategy by introducing an electron-donating methoxy group into DOL, resulting in a non-fluorinated cyclic ether solvent–2-methoxy-1,3-dioxolane (MODOL) (Fig. 11f) [218]. MODOL exhibits high compatibility with Li metal and a weak Li⁺ solvation ability (Fig. 11g, h), leading to an anion-dominated solvation structure in the electrolyte. The MODOL-based electrolyte enables a LFP || Li cell to retain 90% of its capacity after 110 cycles at − 20 °C. Zhao et al. designed a class of anchor-WSE (AWSEs) at conventional salt concentrations by tuning the chain length of polyoxymethylene ether solvents (Fig. 11i) [219]. This strategy effectively mitigates the co-intercalation issue of Gr anodes without compromising ionic dissociation ability. They found that the oxidative stability of AWSEs increased with the elongation of the chain length and the enhancement of the “anchoring effect,” as evidenced by LSV. Among these molecules, dipolyformaldehyde dimethyl ether (DDE) exhibited superior oxidative stability and high interfacial reaction kinetics. As a result, a NCM811 || Gr cell using the DDE-based electrolyte retained 75.86% of its capacity after 400 cycles at − 20 °C. A partially WSE (PWSE) was developed by dissolving 1.3 M LiFSI in a DME/1,2-Bis(1,1,2,2-tetrafluoroethoxy)ethane (TFEE) mixed solvent with a volume ratio of 2:8 [114] (Fig. 12a, b). TFEE was employed to modulate the local environment of the electrolyte, weakening the bidentate chelation between Li⁺ and DME and thereby resulting in weak coordination with Li⁺, which reduces the desolvation energy barrier at LTs. In addition, the incorporation of lithium fluoromalonato(difluoro)borate (LiFMDFB) and silver nitrate (AgNO₃) further promotes the formation of a stable interface.

Recent studies have demonstrated that ion–dipole interactions can modulate the solvation environment of ether-based electrolytes, thereby enhancing their oxidative stability and LT performance [220]. A non-concentrated, fluorine-free ether electrolyte formulated by dissolving a ternary mixture of LiDFOB, LiPF_6_, and LiBF_4_ in THF was shown to form stable THF–Li⁺–anion complexes through appropriate ion–dipole interactions. These interactions effectively reorder the degradation sequence of electrolyte components, thus significantly improving the oxidative stability of the THF-based electrolyte. As a result, NCM811 || Li cells utilizing this electrolyte exhibit stable cycling performance under − 30 °C and 2.7–4.5 V conditions. Li et al. reported a strategy employing a non-solvating co-solvent to weaken the electron-donating ability of ether solvents. By introducing TTE into a WSE (LiFSI dissolved in a mixed solvent of THF and 2MeTHF), a temperature-insensitive solvated electrolyte (TISE) was obtained. This TISE remains in the liquid state at temperatures as low as − 100 °C. LFP ||Li pouch cells utilizing this TISE exhibited high CR over 150 cycles at both − 20 and − 40 °C [221].

Although WSEs have achieved certain progress, the weak affinity between solvent molecules and Li⁺ leads to insufficient Li salt dissociation, resulting in low ionic conductivity in the bulk electrolyte and even Li salt precipitation at LTs. Furthermore, the poor film-forming ability of weakly solvating solvents makes it difficult to stabilize the electrode–electrolyte interface effectively. In such cases, interfacial stability relies entirely on the Li salt. However, due to the limited solubility of Li salts in weakly solvating solvents, the salts are rapidly depleted during cycling, compromising interfacial stability. Therefore, it remains a significant challenge to simultaneously ensure fast Li⁺ transport in the bulk electrolyte and efficient desolvation at the electrode–electrolyte interface.

#### Nitrile-Based Solvents

**Fig. 13:**
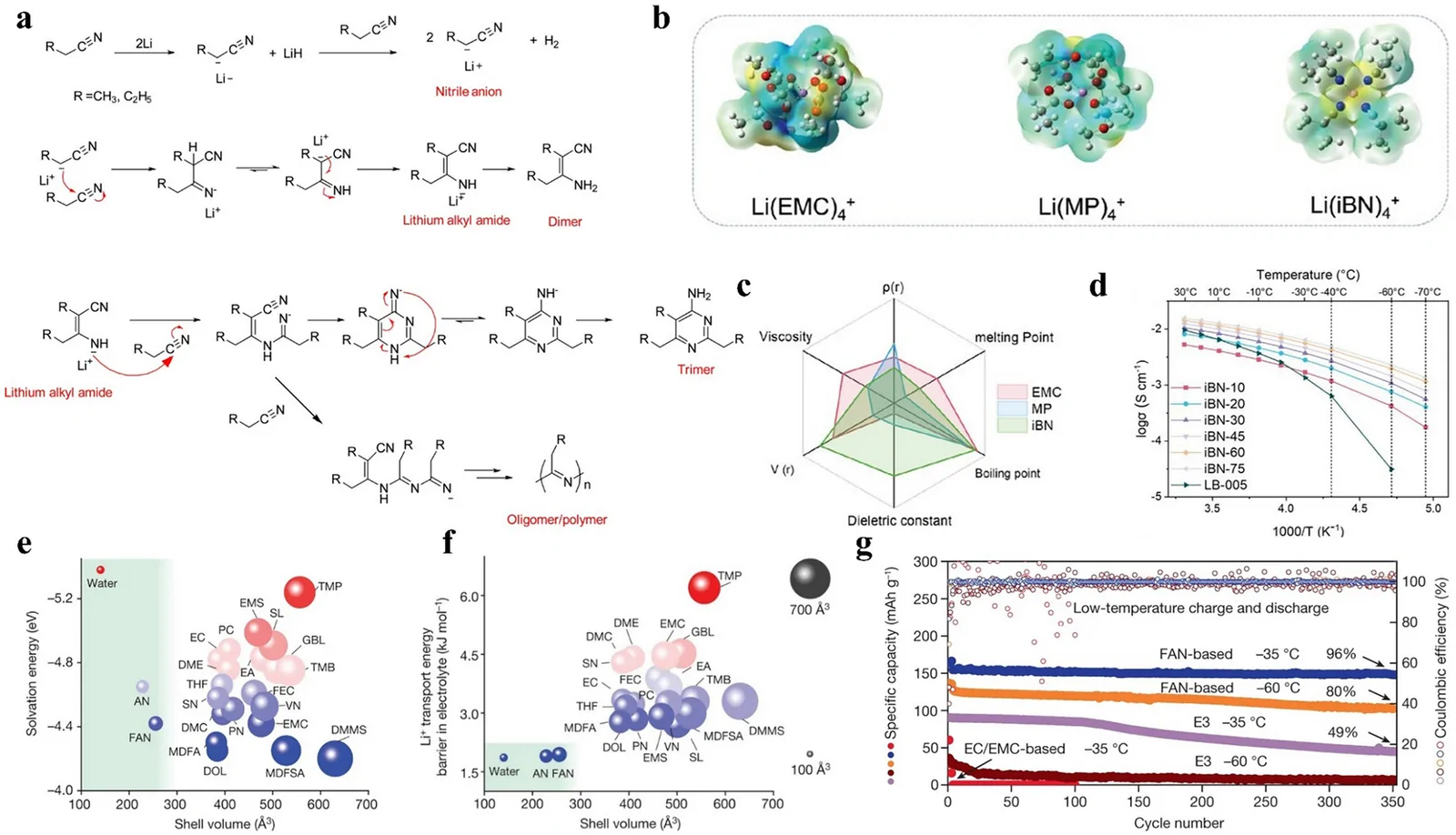
**a** Schematic diagram of the reaction mechanism between Li metal and mononitriles [225]. **b** The optimized tetra-coordination solvation structures of Li^+^ with EMC, MP, and iBN [228]. **c** Radar chart of six important properties of EMC, MP, and iBN as electrolyte cosolvents [228]. **d** Ionic conductivity of various electrolytes [228]. **e** Solvation energy and solvation-shell volume of solvents [229]. **f** Li^+^ transport energy barrier and solvation-shell volume of solvents [229]. **g** Cycling performance of NMC811||Gr cells with different electrolytes in the range 2.8–4.5 V at − 35 °C and − 60 °C [229]

Compared with carbonate and ether solvents, nitrile-based solvents exhibit higher molecular polarity and DC, making them distinctive co-solvents in LIB electrolytes [222, 223]. For instance, acetonitrile (AN), propionitrile (PN), and isobutyronitrile (iBN) all possess DC greater than 20, and their viscosities at 25 °C are sufficiently low, measured at 0.350, 0.389, and 0.456 mPa s, respectively [224]. However, nitrile compounds exhibit an initial reduction potential of around 2 V, which can trigger premature electrolyte decomposition during the reduction process and accelerate Gr exfoliation. Moreover, nitrile-based solvents tend to undergo side reactions with metallic Li, resulting in a series of organic byproducts that hinder the formation of a dense and stable SEI on the Li surface, thereby compromising the LT performance of LMBs. Lee et al. discovered that mononitriles undergo side reactions with metallic Li, potentially leading to the formation of dimers, trimers, and oligomeric/polymeric byproducts (Fig. 13a) [225]. They proposed a reaction mechanism to explain the instability of mononitriles toward Li. Specifically, the α-hydrogen of nitriles (pKa ∼ 30) can be easily abstracted by bases, forming Li nitrile and Li hydride. The Li hydride can further react with another mononitrile molecule to produce hydrogen gas and additional Li nitrile. The resulting nitrile anion acts as a nucleophile and attacks the nitrile carbon of another mononitrile molecule, initiating a nucleophilic addition reaction to form a lithiated alkylamine intermediate. This intermediate undergoes rearrangement to yield a dimer, which may further attack additional nitrile molecules, eventually forming conjugated oligomers and polymers. Due to their high solubility in organic solvents, these byproducts fail to form a stable protective layer on the Li metal surface, resulting in continued reactions and further decomposition of the mononitriles. In contrast, dinitrile molecules, containing two cyano groups, can form cross-linked structures and generate insoluble products that deposit on the lithium surface as a protective layer, thereby effectively suppressing continuous reactions with lithium. Nevertheless, a key drawback of dinitrile and polynitrile solvents lies in the need to extend the carbon chain length as the number of –CN groups increases, which leads to higher molecular viscosity and hampers lithium salt dissociation. Additionally, the increased steric hindrance may reduce the molecular coverage on the cathode surface, limiting ‒CN adsorption and the formation of a protective interphase, ultimately compromising electrochemical performance [226]. Although high-concentration nitrile-based electrolytes can deliver excellent electrochemical performance, they hinder Li^+^ diffusion and transport, resulting in increased viscosity, limited LT performance, and higher costs. Using nitrile solvents in combination with carbonate-based solvents as co-solvents is considered a viable strategy, as it maintains high LT capacity retention even at low Li salt concentrations. The high DC of nitriles weakens the binding energy between Li^+^ and solvent molecules, facilitating rapid desolvation and enabling faster charge transfer.

Wang et al. formulated electrolytes using nitrile solvents, such as AN, PN, and butyronitrile (BN) as cosolvents. Owing to their low viscosities, low freezing points, and ability to enhance ionic conductivity, LIBs based on these electrolytes exhibited significantly improved charge/discharge performance at − 20 °C, while also maintaining excellent performance even under a high rate of 3 C [227]. Compared with EMC and MP, iBN exhibits the weakest interaction with Li^+^ (Fig. 13b, c). When used as a co-solvent in carbonate-based electrolytes, it enables the electrolyte to achieve a conductivity of 1.152 mS cm^−1^ at − 70 °C (Fig. 13d). This allows the LCO || Gr cell to retain 68.7% of its room-temperature discharge capacity at − 70 °C, while also exhibiting stable cycling performance at − 40 °C [228]. Fluorination of nitrile solvents is also an important strategy for enhancing their electrochemical performance. After fluorination, nitrile solvents exhibit further reductions in freezing point and improved tolerance to high voltages, thereby broadening the operating temperature and voltage windows of the electrolyte. Fan et al. designed a novel electrolyte using fluoroacetonitrile (FAN) and 1.3 M lithium bis(fluorosulfonyl)imide, which exhibited an ionic conductivity of 40.3 mS cm^−1^ at room temperature and 11.9 mS cm^−1^ at − 70 °C (Fig. 13e, f) [229]. The FAN solvent, which combines high ionic conductivity, low solvation energy, and a small molecular size, can extract Li^+^ from the primary solvation sheath, forming a fast ion-conducting ligand channel that reduces the energy barrier for ion transport. Moreover, FAN allows anions to enter the primary solvation shell of Li^+^, leading to the formation of an inorganic-rich interfacial layer. Benefiting from these features, a NMC811||Gr cell achieved a capacity retention of 80% after 350 cycles at − 60 °C (Fig. 13g).

Table 3 summarizes the electrochemical performance of various electrolyte systems constructed with different solvents at LTs. Rational solvent selection not only helps reduce system viscosity, enhance Li salt solubility and ionic conductivity, but also enables modulation of the Li⁺ solvation structure and reduction of the Li⁺ desolvation energy barrier at LTs, thereby improving the LT performance of LIBs. Therefore, precise optimization of the ratios among multiple solvents is essential to achieve an optimal balance of electrochemical properties. In addition, the development of novel electrolyte systems—such as high-entropy electrolytes (HEEs)—with both high ionic conductivity and interfacial stability at low temperatures will become a key research direction. This can be achieved through multidimensional strategies including molecular design, high-throughput screening, and AI-assisted optimization.

**Table 3:** Electrochemical performance of various electrolyte formulations regulated by solvent tuning strategies

Electrolyte formulation	Cell type	CR at LT (current density)	Cycle number	Refs
1.5 M LiFSI-MP/FEC (9: 1 by vol)	LFP || Gr	88.0% at − 30 ℃ (0.1 C)	100	[159]
1.1 M LiFSI-MA/FB (1: 4: 5 by molar)	LCO || Gr	90% at − 20 ℃ (1 C)	1000	[164]
0.9 M LiFSI + 0.3 M LiDFOB-FEC/DFEA/TTE (2: 4: 4 by weight)	LCO || Gr	82.2% at − 20 ℃ (0.2 C)	200	[148]
0.5 M LiFSI-EDFA/FEC (9: 1 by vol)	NCM811 || Gr	≈100% at − 20 ℃ (0.2 C)	40	[144]
2.5 M LiBF4-DOL/DME (7:3 by vol) with 2 vol.% Si-NCO	LCO || Li	80% at − 40 ℃ (0.33 C)	150	[192]
1 M LiFSI-BTFE/DME (5:1 by molar)	NCM811 || Li	99.08% at − 40 ℃ (0.2 C)	100	[204]
1 m LiFSI-TFMP/DME (7: 1 by vol)	NCM811 || Li	≈100% at − 20 ℃ (0.2 C)	200	[205]
1 M LiFSI-DEE/TFM (1: 2 by vol)	FeS_2_|| Li	83.1% at − 20 ℃ (0.1 C)	80	[230]
1 M LiFSI-IZ/TTE (1: 4.5 by vol) with 10 vol% FEC	SPAN || Li	99.85% at − 30 ℃ (0.1 C)	50	[231]
1 M LiFSI-EMC/FEC / DTF (1.5: 1.5: 7 by vol)	NMC811 || Li	93% at − 40 ℃ (0.2 C)	100	[206]
1 M LiFSI-DMM	SPAN || Li	63.8% at − 40 ℃ (0.1 C)	120	[211]
1 M LiFSI + 3 wt% LiNO_3_-THP/FEC (95:5 by vol)	NMC811 || Li	87% at − 40 ℃ (0.2 C)	100	[215]
LiFSI-CPME (1:2 by molar)	LFP || Li	90% at − 20 ℃ (0.5 C)	400	[217]
1 M LiFSI + 0.3 M LiNO_3_-MODOL	LFP || Li	90% at − 20 ℃ (0.2C)	110	[218]
3 M LiFSI-DE	NCM811 || Gr	75.86% at − 20 ℃ (0.2C)	400	[219]
1.5 M LiFSI-MixTHF/TTE	LFP ||Li	≈100% at − 20 ℃ (0.2C)	150	[221]
1 M LiFSI-HEX/HME (1: 1 by vol)	SPAN || Li	93.7% at − 30 ℃ (0.1C)	100	[232]
1 M LiFSI-MTHF/THF (6: 1 by vol) + 1 wt% LiNO_3_	CoSeO_x_|| Li	84% at − 20 ℃ (400 mA g^−1^)	100	[233]
1.3 M LiFSI- DME/TFEE (2:8 by vol) + 1% LiFMDFB + 0.05% AgNO3	LCO || Li	79.3% at − 20 ℃ (0.2 C)	300	[114]

### Film-Forming Additives

**Fig. 14:**
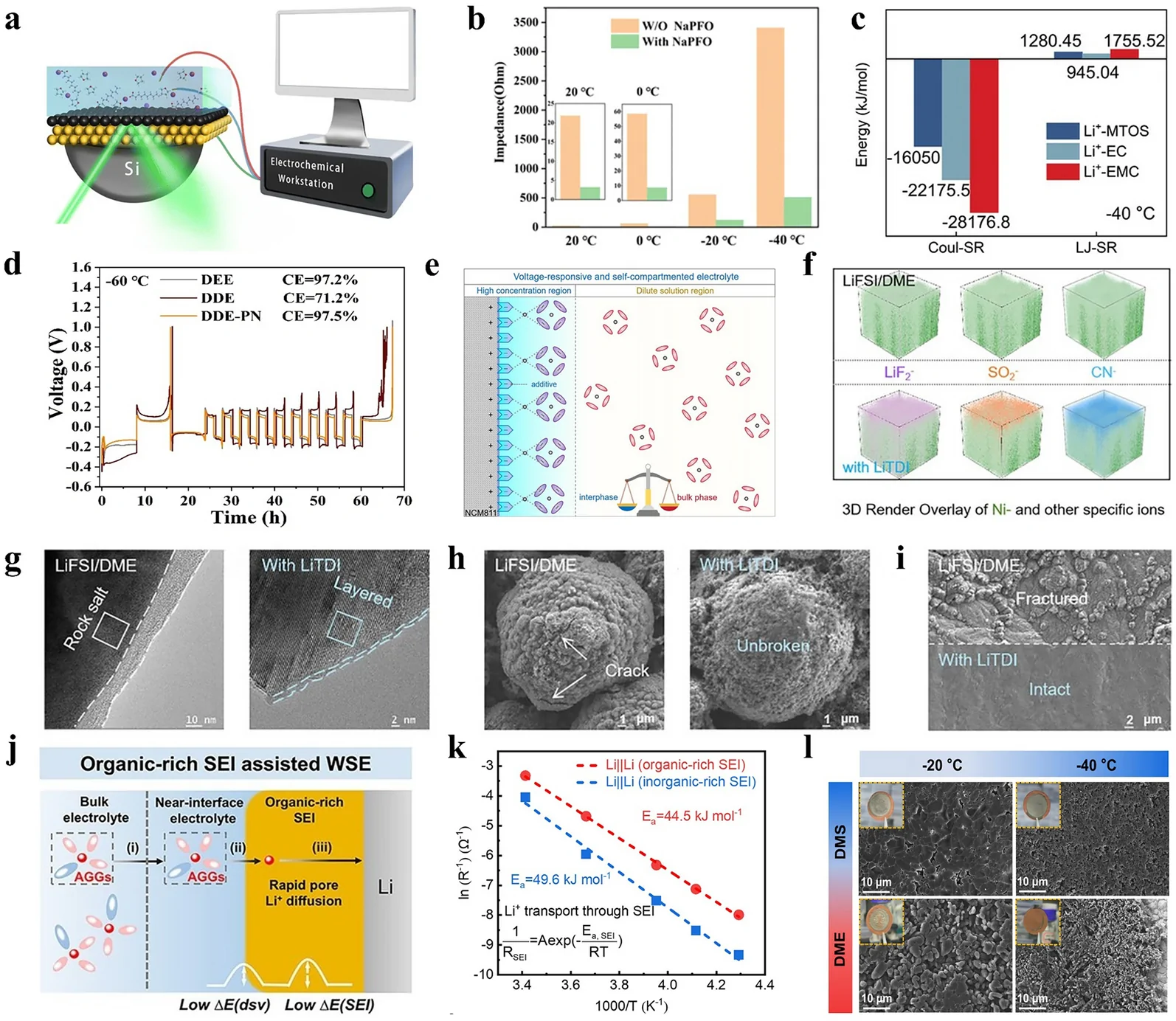
**a** Schematic diagram of an in-situ EC-ATR-SEIRAS cell [239]. **b** Impedance at different temperatures [239]. **c** Interaction energy between Li^+^ and solvents [241]. **d** CE evaluated by Li||Cu half cells at − 60 °C [242]. **e** Schematic illustration of the LiTDI-based electrolyte balancing ionic conductivity and stabilizing the CEI interface [243]. **f** 3D visualization of TOF–SIMS [243]. **g** High-resolution TEM images of the NCM811 cathode after cycling in two different electrolytes [243]. **h** SEM images of the NCM811 cathode after cycling in two different electrolytes [243]. **i** SEM images of the aluminum current collector behind the NCM811 cathode [243]. **j** Schematic illustration of charge transport processes in organic-rich SEI-assisted WSE [246]. **k** Comparison of the activation energy for Li^+^ diffusion through the SEI [246]. **l** Li deposition morphology obtained by SEM [246]

Studies have shown that the desolvation process of Li^+^ involves not only the electrolyte but also the SEI, which plays a crucial role in Li⁺ desolvation. Moreover, it has been demonstrated that anion-derived SEI directly facilitates the desolvation of Li^+^ [234]. For example, inorganic SEI components such as Li_2_CO_3_, LiF, Li_3_P, and Li_3_PO_4_ can facilitate Li⁺ desolvation within the SEI by weakening the interaction between Li⁺ and solvent molecules [235–237]. Therefore, it is necessary to introduce additives with excellent film-forming capabilities to construct a robust interfacial layer with high ionic conductivity and mechanical stability. The primary function of film-forming additives is to undergo preferential reduction (or oxidation) over the main solvent during the initial charge–discharge activation process, thereby forming a stable interfacial layer (SEI or CEI) on the anode (or cathode) surface. Consequently, these additives must possess specific energy level characteristics: those intended for SEI formation on the anode should have a lower LUMO energy level to facilitate reduction at higher potentials, whereas those designed for CEI formation on the cathode should exhibit a higher HOMO energy level to enable preferential oxidation at lower potentials [238]. For instance, 1,3-propane sultone (PS), when used as an additive in an electrolyte composed of 1 M LiPF₆ dissolved in a mixed solvent of EA and FEC at a 10:1 volume ratio, exhibits high ions conductivity, low viscosity, and excellent chemical stability. PS contributes to the formation of a CEI with favorable mechanical stability and flexibility, as well as an SEI rich in inorganic components [24]. An 'electric field-assisted self-assembled interphase' strategy has been proposed to regulate the microenvironment at the SEI by introducing sodium perfluorooctanoate (NaPFO) as an additive. As shown in Fig. 14a, in situ electrochemical attenuated total reflectance surface-enhanced infrared absorption spectroscopy (EC-ATR-SEIRAS) reveals that NaPFO enables the self-assembly of interfacial molecular layers under an applied electric field, facilitating the formation of a stable and compact SEI. Compared to the electrolyte without NaPFO, the NaPFO-containing electrolyte exhibits lower interfacial and bulk resistances at LTs (Fig. 14b), thereby accelerating Li⁺ transport and the desolvation process at the electrode interface. Owing to this approach, a NMC811 || Li pouch cell achieves an energy density of 122 Wh kg⁻^1^ even at − 85 °C [239].

In recent years, organosilicon compounds featuring Si–C and Si–O bonds have attracted considerable attention in LT electrolyte systems due to their excellent DC, moderate polarity, and strong film-forming capabilities. Their significant potential in improving the LT performance of LIBs positions them as promising candidates for next-generation high-performance electrolyte materials [240]. Yan and collaborators demonstrated, through MD simulations, nuclear magnetic resonance (NMR), and Raman spectroscopy, that the Si–O conjugation effect in methyltrimethoxysilane (MTOS) reduces the electron-donating ability of oxygen atoms. This, in turn, weakens the interaction between Li⁺ and the solvent (Fig. 14c), thereby promoting the formation of anion-derived solvation structures. Moreover, the solvation shell jointly formed by MTOS and the FSI⁻ anion contributes to the construction of an interfacial layer enriched in LiF and Si–O species, which significantly reduces interfacial resistance and enhances ion transport. As a result, the NCM811|| Gr pouch cell employing this electrolyte retains 88.85% of its initial capacity after 100 cycles at − 20 °C under a 0.2 C rate [241]. Liu et al. reported a film-forming additive, perfluoroalkylsulfonyl quaternary ammonium nitrate (PQA-NO₃, noted as PN) [242]. The cation (PQA⁺) can react in situ with lithium metal to form an inorganic-rich and stable SEI, thereby enhancing Li⁺ diffusion within the SEI. Meanwhile, the anion (NO₃⁻) promotes the formation of an anion-rich solvation structure and reduces the interaction between Li⁺ and solvent molecules. An electrolyte, referred to as DDE-PN, was prepared by adding 0.1 M PN to 1.0 M LiFSI dissolved in a DEE and DME mixture with a volume ratio of 9:1. This electrolyte enabled the Li||Cu cell to achieve a high CE of 97.5% at − 60 °C, indicating excellent interfacial transport kinetics and outstanding lithium plating/stripping reversibility (Fig. 14d). Moreover, the NCM811||Li full cell employing this electrolyte maintained 93.3% of its initial capacity after 100 cycles at − 60 °C. Lithium 4,5-dicyano-2-(trifluoromethyl) imidazol-1-ide (LiTDI) has been widely employed as a film-forming additive due to its unique molecular structure. For example, a self-domain electrolyte design strategy was reported, in which LiTDI was introduced as an additive into a 1 M LiFSI in DME electrolyte [243]. LiTDI facilitates the formation of an anion-dominated solvation structure at the cathode surface, thereby constructing a stable and low-impedance CEI; meanwhile, the bulk electrolyte retains a solvent-dominated solvation structure to ensure rapid Li⁺ transport (Fig. 14e). TOF–SIMS analysis revealed that the CEI was primarily composed of inorganic species, such as LiF and LiSOₓ (Fig. 14f). High-resolution transmission electron microscopy (TEM) and scanning electron microscopy (SEM) analyses revealed that the LiTDI-based electrolyte enabled the formation of a uniform and dense CEI on the NCM811 cathode surface and effectively suppressed the corrosion of the aluminum current collector by the electrolyte (Fig. 14g-i). The NCM811 || Li cell using this electrolyte exhibited a high CR of 96.6% after 700 cycles at − 20 °C under a 0.5 C charge/discharge rate, with an average CE of 99.2%.

Current research primarily focuses on designing inorganic-rich SEI to enhance the LT and high-voltage performance of LMBs [235, 236]. However, recent studies have suggested that constructing inorganic-rich SEIs may not be the optimal strategy for enhancing the LT performance of LMBs, as such SEIs fail to effectively reduce interfacial resistance [244, 245]. Specifically, highly polar inorganic-rich SEIs can attract Li⁺ ions, thereby minimizing Li⁺–solvent pairing. However, this characteristic may also promote the incorporation of additional solvent molecules into the interfacial solvation structure, potentially enhancing Li⁺–solvent coordination. These opposing effects may, in certain electrolyte systems, hinder the desolvation process and consequently impair the interfacial transport kinetics of Li⁺. In contrast, due to their weaker polarity, organic components are less likely to attract solvent molecules, thereby helping to preserve anion-rich solvation structures in WSE systems. Yin et al. dissolved 1.5 M LiFSI in dimethyl dimethoxy silicane (DMDMS) to prepare an organosilicon electrolyte, which was applied to NCM811 || Li cells. This system facilitated the formation of an organic SEI rich in Si–O bonds [246]. It was demonstrated that the organic-rich SEI, compared to the anion-derived inorganic-rich SEI, possesses lower resistance at the electrode interface, thereby significantly enhancing the LT cycling performance of LMBs (Fig. 14j). Experimental results revealed that, at LTs, the Li⁺ desolvation energy barrier and the activation energy for Li⁺ diffusion through the SEI on LMA with organic-rich SEIs were significantly lower than those associated with inorganic-rich SEI (Fig. 14k). Moreover, SEM observations confirmed that the DMDMS-based electrolyte enabled uniform Li deposition (Fig. 14l).

Table 4 summarizes the electrochemical performance of the electrolyte systems designed with various film-forming additives under LT conditions. Rational selection of film-forming additives can effectively promote the formation of structurally robust and highly ion-conductive SEI and CEI layers at LTs. This enhances Li⁺ diffusion kinetics at the interfaces, thereby improving the LT performance of LIBs. Although film-forming additives demonstrate promising effectiveness in enhancing LT performance, their rational screening still faces multiple challenges. Currently, there is a lack of systematic screening methodologies, and existing strategies predominantly rely on empirical or trial-and-error approaches, resulting in low screening efficiency and limited scalability. Furthermore, their reaction mechanisms under LT conditions remain unclear, and critical parameters such as the composition of interfacial products have not been fully elucidated. Therefore, integrating AI technology is essential to screen optimal film-forming additives and accelerate experimental workflows. Concurrently, advanced characterization techniques combined with theoretical computations should be employed to elucidate their interfacial evolution mechanisms.

**Table 4:** Electrochemical performance of various electrolyte formulations regulated by additive tuning strategies

Electrolyte formulation	Cell type	CR at LT (current density)	Cycle number	Refs
1.0 M LiPF6-EA/FEC (10: 1 by vol) + 2 vt% PS	NCM811 || Li	≈80% at − 30 ℃ (0.1C)	200	[24]
1.0 M LiFSI-THF + 0.5 wt% NaPFO	NCM811 || Li	72% at − 60 ℃ (0.1 C)	80	[239]
1.5 M LiFSI-MTOS	NCM811 || Gr	88.85% at − 20 ℃ (0.2 C)	100	[241]
1.0 M LiFSI-DEE: DME (9:1 by vol) + 0.1 M PN	NCM811 || Li	93.3% at − 60 ℃ (0.05 C)	100	[242]
1.0 M LiPF6-EC/EMC DMC (1:1:1 by vol) + 2.5 wt% LiDFP	NCM523 || LPO-Gr	86.6% at − 10 ℃ (0.1 C)	300	[236]
1.0 M LiFSI-DME + 0.2 M LiTDI	NCM811 || Li	96.6% at − 20 ℃ (0.5 C)	700	[243]
1.5 M LiFSI-DMDMS	NCM811 || Li	95.2% at − 40 ℃ (0.1 C)	180	[246]
LiFSI + LiODFB (7: 3 by molar)-MA/FEC (4: 1 by vol) with 0.15 mol kg^−1^ LiNO_3_	NCM523 || Li	84% at − 40 ℃ (0.2 C)	150	[247]
2.4 M LiFSI-FEC/DMC (3: 7 by vol)	LFP| | Li	≈98% at − 20 ℃ (0.2 C)	400	[237]
1 M LiPF_6_-EC/EMC (1: 1 by vol) with 0.2 wt% LiNO_3_ + 2 wt% FS + 5 wt% PBF	LFP| | Li	89.4% at − 40 ℃ (0.5 C)	200	[248]

### Gel Polymer Electrolytes (GPEs)

**Fig. 15:**
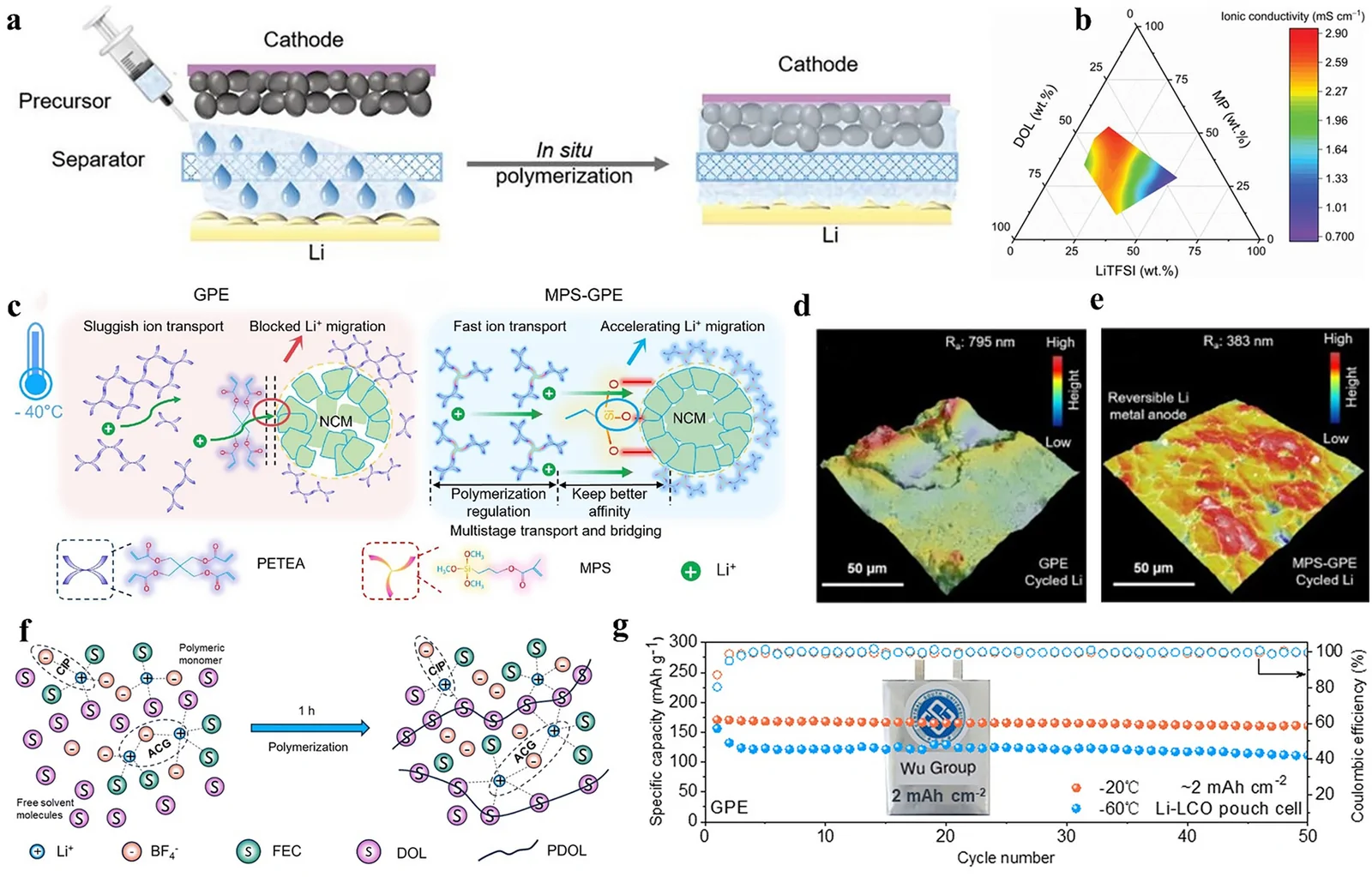
**a** Schematic illustration of integrated battery production via in situ polymerization [256]. **b** Ionic Conductivity gradient of the electrolyte on the ternary phase diagram [256]. **c** Interfacial schematic of GPE and MPS-GPE at LT [257]. SEM 3D surface roughness reconstruction of cycled Li anode with **d** GPE and **e** MPS-GPE [257]. **f** Schematic illustration of interactions between components during the polymerization process [258]. **g** Cycling performance of LCO || Li pouch cell [258]

GPEs, owing to their unique advantages, are widely regarded as promising candidates for next-generation LMBs. First, GPEs can effectively mitigate the safety risks associated with leakage and explosion of organic liquid electrolytes, thereby enhancing battery safety [249]. In addition, compared to conventional liquid electrolytes, GPEs exhibit a higher Young’s modulus, which enables superior suppression of Li dendrite growth, thus extending battery lifespan and improving cycling stability [250]. More importantly, GPEs hold great potential for application in flexible batteries for future smart wearable devices. However, conventional GPEs typically exhibit poor ionic conductivity and high desolvation energy at LTs, rendering them unsuitable for operation in LT LIBs [251, 252]. Fortunately, recent research has made significant progress in material design and structural optimization, such as adjusting the polymer backbone and solvation structure [253–255]. These advances have significantly improved the electrochemical performance of GPEs and enhanced their potential for application in LT LIBs. For example, Ciucci et al. reported a GPE synthesized via in situ ring-opening polymerization of DOL, in which the ratio of solvent (MP), Li salt (LiTFSI), additive (FEC), and initiator (LiPF₆) was carefully optimized (Fig. 15a, b) [256]. The fluorine-rich interfacial layer induced by FEC significantly enhanced interfacial stability, while MP, with its low freezing point of − 88 °C, effectively improved ionic transport at LTs. A NCM811 || Li cell employing this GPE delivered a discharge capacity of 109 mAh g⁻^1^ after 100 cycles at − 20 °C. The incorporation of methacryloxypropyltrimethoxysilane (MPS) into the GPE enhances the interfacial Li⁺ transport kinetics under LT conditions (Fig. 15c). MPS-GPE enables more uniform Li deposition than GPE, which helps suppress dendrite formation (Fig. 15d, e). A NCM811 || Li cell using this electrolyte exhibited a high CE of 99.98% at − 40 °C [257]. In addition, Chen et al. prepared a GPE for LT operation of LMBs by dissolving LiBF_4_ in a mixed solvent of DOL and FEC (Fig. 15f) [258]. This GPE exhibited a high ionic conductivity of 0.53 mS cm^−1^at − 20 °C. During the Li⁺ solvation process, BF₄⁻ played a dominant role, accounting for over 85% of the coordination. As a result, the formed SEI was primarily composed of stable LiF and Li⁺-conductive Li_x_BO_y_F_z_ generated from the reductive decomposition of BF₄⁻ and FEC, thereby enhancing the Li⁺ transport kinetics. The LCO|| Li pouch cell employing this GPE retained over 90% of its capacity after 50 cycles at − 60 °C (Fig. 15g). A GPE was synthesized via in situ ring-opening polymerization of DOL within a polypropylene separator, using LiFSI and LiDFOB as co-salts [259]. LiDFOB acted as both a functional additive for interfacial stabilization (forming B-rich CEI) and a polymerization initiator, enabling impurity-free synthesis. The resulting GPE exhibited high ionic conductivity, a Li⁺ transference number of 0.61, and enhanced oxidation stability. The NCM811||Li cell employing this gel polymer electrolyte exhibited stable cycling performance at − 20 °C, maintaining 88.4% of its initial capacity after 120 cycles. Weakly solvating GPE have also attracted considerable attention from researchers. For example, a weakly solvating ether-based GPE was developed by in situ polymerization of 1.0 M LiFSI in DOL [260]. The resulting GPE forms a stable SEI with a 3D desolvation interface, which significantly facilitates Li⁺ desolvation and transport across the interface and within the SEI. This weakly solvating ether-based GPE exhibits high ionic conductivity (5.73 mS cm^−1^ at 25 °C) and excellent LT performance (operable at − 40 °C). Benefiting from the above advantages, the Si–C half-cell employing this GPE delivers a discharge capacity of 113.7 mAh g^−1^ after 100 cycles at − 40 °C under 0.5 C. Zhu and co-workers reported a weakly solvating GPE designed to enhance the LT performance and safety of LMBs. Poly (trifluoroethyl methacrylate) (PTFMA) was employed as the brush-like polymer backbone, while ethyl 3,3,3-trifluoropropionate (FEP) served as a coupling agent [23]. The dual dipole–dipole interaction between FEP and PTFMA led to the enrichment of FEP around the polymer brushes and effectively weakened the coordination between FEP and Li⁺. The electrolyte exhibited ionic conductivities of 4.40 × 10^–4^ and 1.03 × 10^–4^ S cm^−1^ at 25 and − 40 °C, respectively, with a Li⁺ transference number of 0.83 and an electrochemical stability window up to 5.05 V. In addition, it demonstrated excellent flame-retardant properties. Owing to these advantages, the GPE enabled stable operation of LMBs across a wide temperature range from − 30 to 80 °C.

Table 5 summarizes the electrochemical performance of various GPEs under LT conditions. In summary, compared to traditional liquid electrolytes, the improved GPEs combine good mechanical flexibility, low interfacial impedance, and excellent dendrite suppression capability, enabling it to effectively enhance the LT performance and safety of LIBs. Despite this, the design of most current GPE systems still heavily relies on complex electrolyte formulations, involving the synergistic combination of multi-salt systems, functional additives, and significant amounts of low-melting-point solvents. While these strategies have significantly improved LT performance to some extent, they also introduce practical issues such as increased costs and complex fabrication processes. Particularly under extreme LTs of − 40 °C and below, their practical application continues to face considerable challenges. Therefore, future research must urgently focus on regulating solvation structures and optimizing polymer matrices to develop streamlined, high-efficiency GPE systems with balanced performance. This approach will achieve holistic optimization of ionic conductivity, interfacial stability, and manufacturability under LT conditions.

**Table 5:** Electrochemical performance of various GPEs

Electrolyte formulation	Cell type	CR at LT (current density)	Cycle number	Refs
1.0 M LiTFSI + 0.5 M LiPF_6_-MP/DOL/FEC (5: 4: 1 by vol)	NCM811 || Li	95.6% at − 20 ℃ (0.2 C)	100	[256]
1 M LiPF₆-EA/MP/FEC (4:5:1 by vol) + 5 wt% PETEA/2 wt% MPS/0.3 wt% AIBN	NCM811 || Li	98% at − 40 ℃ (0.1 C)	100	[257]
2 M LiBF4- FEC: DOL (1: 9 by vol)	LCO || Li	96% at − 20 ℃ (0.2 C)	350	[258]
0.6 M LiFSI + 0.4 M LiDFOB in DOL	NCM811 || Li	88.4% at − 20 ℃ (0.5 C)	120	[259]
In situ polymerization liquid precursor electrolyte (1 M LiFSI in DOL)	Si–C| | Li	82.7% at − 40 ℃ (0.5 C)	100	[260]
1.0 M LiTFSI–FEP + 5 wt% FEC + 20 wt% TFMA + 1 wt% PEGDA + 0.5 wt% AIBN	NCM811 || Li	97.1% at − 20 ℃ (37.6 mA g^−1^)	200	[23]

### AI-Assisted Design of LT Electrolytes

Within the chemical space, numerous substances can be utilized as components for LT electrolytes, each characterized by distinct molecular structures, physical properties, and electrochemical stability. Selecting electrolyte components that simultaneously exhibit excellent LT performance, safety, and oxidative stability from the vast chemical space remains a major challenge in the design of LT electrolytes. Despite years of effort by researchers to develop advanced electrolytes through molecular design, a large portion of the chemical space remains unexplored, and the relationship between the molecular structure of electrolytes and their performance in practical battery systems is still not well understood. High-throughput methods can significantly accelerate the electrolyte design process [261, 262]. For instance, group contribution (GC) [263] and MD simulations [264, 265] have proven to be effective tools for expediting electrolyte development. However, these methods often suffer from limited generalizability, being restricted to specific systems or single properties, and typically require extensive human intervention.

**Fig. 16:**
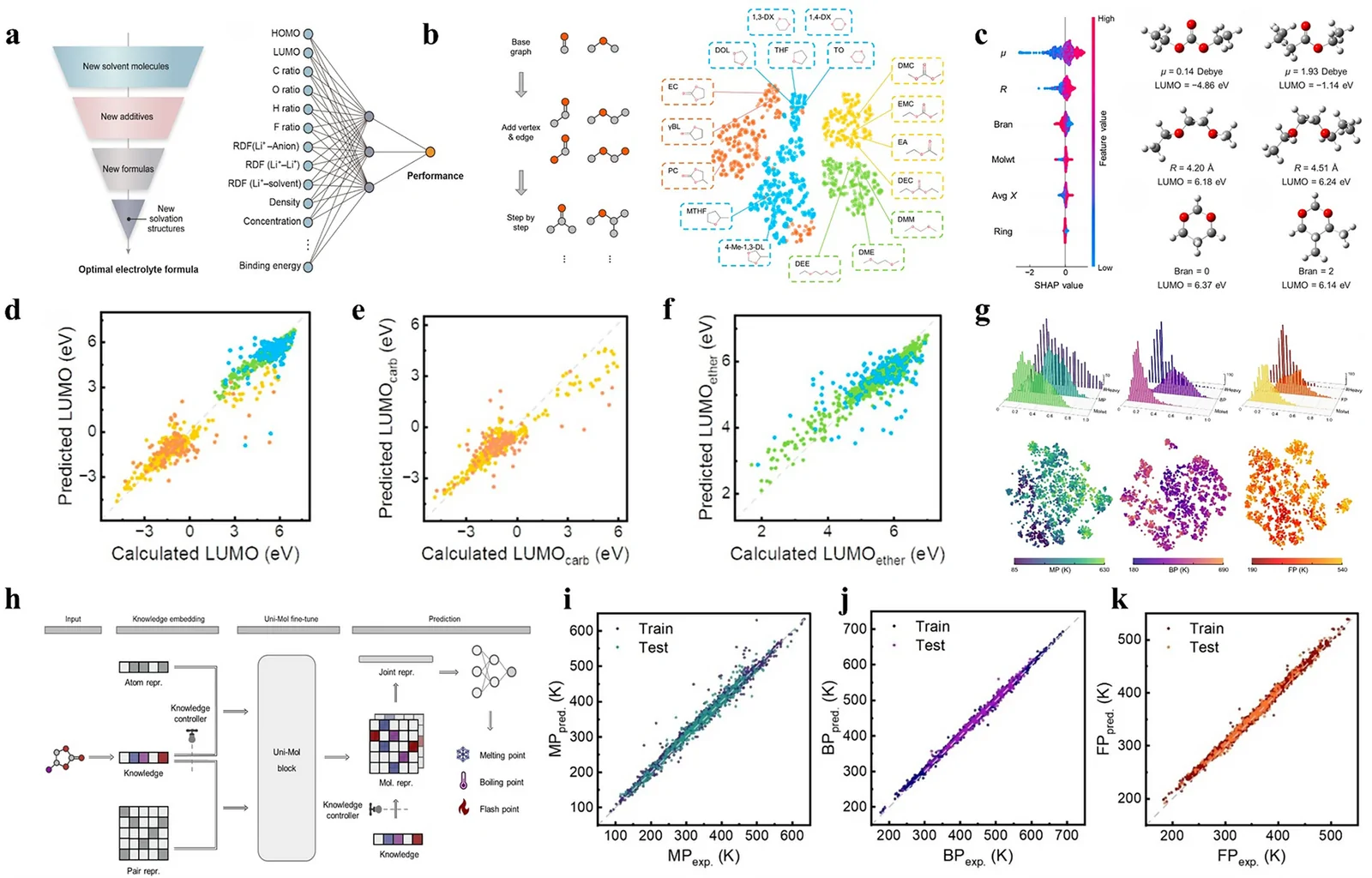
**a** AI-guided optimization and design of LT electrolytes [273]. **b** Construction of the solvent molecule database. **c** Interpretable ML for predicting the LUMO energy levels of ion–solvent complexes [269]. Results of RF model prediction for **d** all 1399 molecules, **e** carbonyl compounds, and **f** ethers [269]. **g** Frequency distribution and visualization analysis of melting Points, BPs, and FPs [290]. **h** Schematic diagram of a knowledge-based learning model [290]. Prediction results of **i** melting points, **j** BPs, and **k** FPs [290]

The rapid advancement of AI, particularly machine learning (ML) techniques, offers significant advantages over traditional manual analysis in handling large-scale, high-dimensional data, making it an efficient tool for extracting knowledge from a vast array of solvent molecules [266–272]. By leveraging AI techniques, statistical correlations derived from large datasets (including Li salts, solvents, additives, and their formulations) can be utilized to guide the prediction of novel solvent molecules, new additives, innovative formulations, and new solvation structures, thereby optimizing electrolyte design. Moreover, AI can also uncover statistical descriptors related to the overall properties of electrolytes (Fig. 16a) and establish correlation models between the physicochemical properties of electrolytes and battery performance, enabling accurate prediction and optimization of electrolyte performance [273]. Stacey F. Bent and her collaborators trained a ML model using a database of 150 Li||Cu cell CEs to predict and optimize various electrolytes suitable for LMA. Their findings revealed that the solvent oxygen ratio (sO) was the most critical feature, with electrolytes exhibiting lower sO values achieving higher CEs. Furthermore, the model was employed as an auxiliary tool to design a novel fluorine-free electrolyte, resulting in a CE as high as 99.70% [268].

The integration of key electrolyte characteristics such as thermal conductivity [274], electrical conductivity [275, 276], and ion–solvent coordination energy [277] as critical descriptors into ML models has received significant research attention for guiding the design of high-performance electrolytes. Electrical conductivity is a key indicator for evaluating the LT performance of electrolytes. However, the relationship between electrolyte conductivity and temperature exhibits strong nonlinearity, making it extremely challenging to accurately predict conductivity across a wide temperature range [29]. The least squares support vector machine (LSSVM) was employed to establish a nonlinear quantitative structure–property relationship for predicting the electrical conductivity. Trained on 783 samples, the LSSVM model achieved a mean absolute relative deviation of less than 1.9% on an independent test set comprising 97 experimental data points [278]. Bamgbopa et al. employed graph neural networks (GNNs) to develop a data-driven model for predicting the conductivity of various types of ionic liquids [279]. Structural features, molecular features, and combined features were extracted from a dataset of 2,684 ionic liquids to train the GNN model. The model demonstrated superior predictive performance when trained on combined features, achieving a mean absolute error (MAE) of 0.470. This also validates that integrating theoretical chemistry or MD simulations with ML facilitates more accurate predictions of ionic liquid conductivity. Furthermore, the accurate prediction of the ESP [280], thermal conductivity [281], and ion coordination energy [282] of electrolyte solvents using ML facilitates the design of high-performance electrolytes.

Driven by the rapid advancement of explainable ML, research methodologies have shifted from purely data-driven paradigms to knowledge discovery frameworks with high interpretability [283, 284]. Notably, Shapley additive explanations (SHAP), a representative interpretable algorithm, have gained substantial research attention. In SHAP, game theory is applied to conduct comprehensive analyses of variable importance in ML predictions [285, 286]. By utilizing feature contribution values calculated through SHAP, researchers can systematically identify key molecular properties such as solvation energy, electrochemical window, and viscosity that critically determine the performance of LT electrolytes. A novel data-driven methodology integrating graph theory algorithms with interpretable ML models has recently been proposed to elucidate the reduction stability mechanisms of ion–solvent complexes in LMB electrolytes and guide molecular design of advanced electrolytes (Fig. 16b, c) [269]. A comprehensive database of potential solvent molecules was systematically constructed using graph theory algorithms. The random forest (RF) model demonstrated optimal performance in predicting the LUMO energy of 1,399 solvent molecules, achieving a MAE of 0.68 eV (Fig. 16d–f). Further interpretability analysis based on SHAP revealed that dipole moment (*μ*) and molecular radius (*R*) are critical descriptors governing LUMO energy levels.

**Fig. 17:**
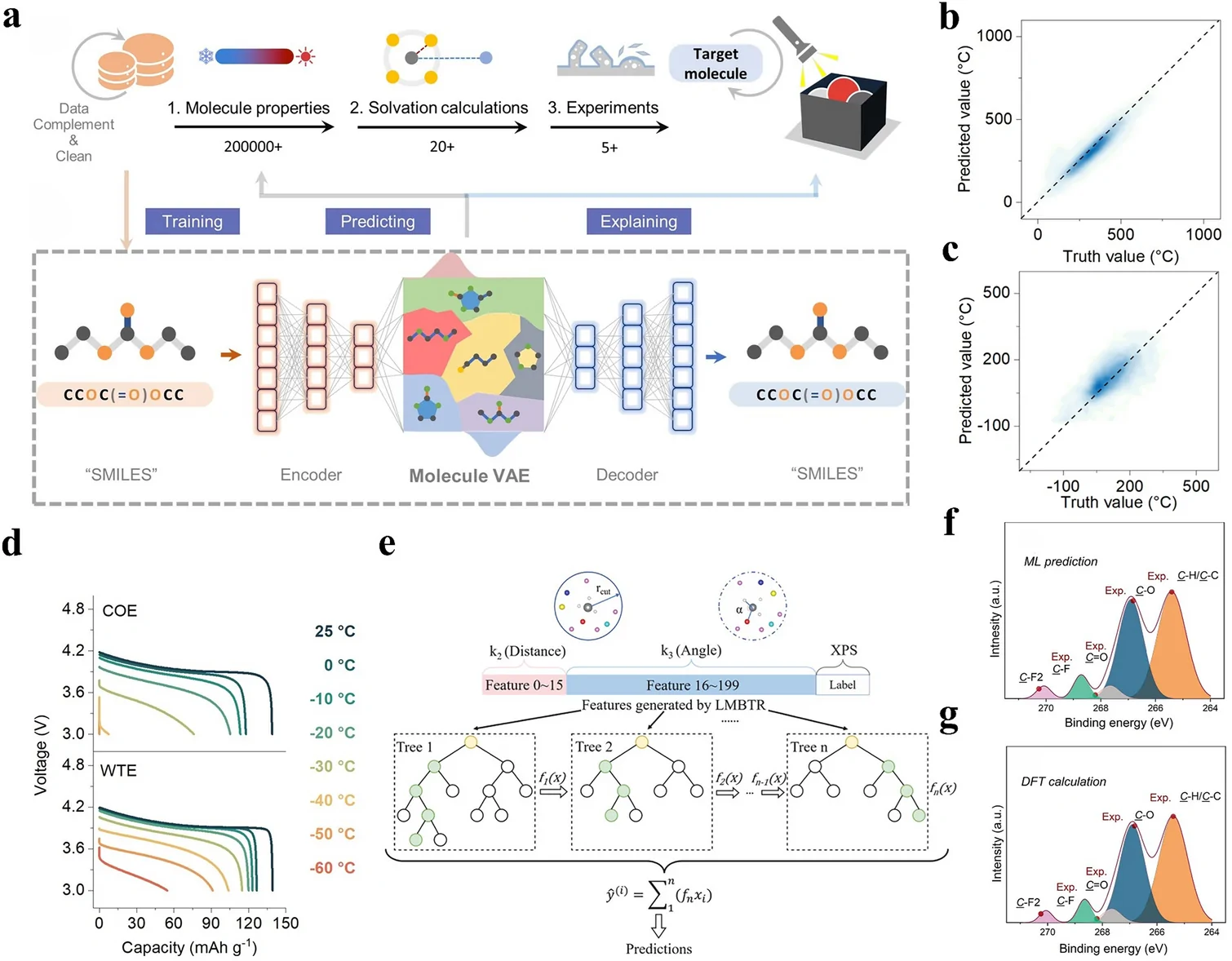
**a** Framework for AI-assisted electrolyte design [291]. Prediction results of the Molecule VAE model for **b** BP and **c** MP [291]. **d** LT discharge profiles of LCO || Li batteries at 0.1C current density (1C = 140 mA g^−1^) [291] **e** Scheme of XPS prediction using ML model [292]. XPS predicted from **f** ML model and **g** DFT calculations [292]

The LT performance of electrolytes is influenced by factors, such as melting point, boiling point (BP), and flash point (FP) [222, 287–289]. For example, electrolytes designed primarily based on melting point and BP criteria have enabled NCM811 || Gr batteries to operate stably at − 60 °C [108]. However, due to the limited availability of experimental data on the physicochemical properties of electrolytes, previous electrolyte design has primarily relied on trial-and-error approaches [272]. This time-consuming method poses significant challenges in the search for electrolytes that simultaneously exhibit a low melting point, high BP, and high FP, ensuring a wide operating temperature range and enhanced safety. Chen et al. reported a knowledge-based property prediction integration (KPI) framework, which consists of data processing and statistical analysis, interpretability and knowledge discovery, and a molecular property prediction module (Fig. 16g, h) [290]. This framework is designed to guide the molecular design of electrolytes across a wide temperature range. The KPI framework demonstrated high accuracy in predicting molecular properties, exhibiting low MAEs of 10.4, 4.6, and 4.8 K for the prediction of melting point, BP, and FP, respectively (Fig. 16i-k). Additionally, through molecular neighborhood searches and high-throughput screening, the KPI framework can predict potential molecules applicable to electrolytes operating across a wide temperature range. This approach can shorten the development cycle of electrolytes, provide a scientific basis for designing electrolyte systems with superior LT performance. Li et al. reported an approach that combines AI-assisted screening with theoretical calculations to identify LT electrolyte solvents (Fig. 17a) [291]. By training an AI model termed Molecule VAE, they established structure–property relationships encompassing over 200,000 molecular candidates at elemental and functional group levels. The model demonstrated high predictive accuracy on a testing dataset parameterized by BP and melting point, achieving root mean square errors (RMSE) of 61.08 and 84.39 K between predicted and experimental values, respectively (Fig. 17b, c). Through this model and its interpretability module, similarities between cyano groups and halogens in terms of melting point, BP, and DC were revealed. Further, the feasibility of using 3-methoxypropionitrile (MPN), a solvent with high conductivity and excellent anode compatibility, as the primary solvent was validated through experimental studies. The AI-guided design of the MPN-based electrolyte enabled the LCO || Li battery to achieve a discharge capacity of 115 mAh g⁻^1^ at − 30 °C, which corresponds to 82.4% of its room-temperature capacity (Fig. 17d). Constructing a SEI and CEI is essential for improving the LT performance of LIBs. To gain a deeper understanding of the formation mechanisms of SEI/CEI and their evolution under LT conditions, advanced characterization techniques have been extensively employed to investigate their microstructures and chemical compositions. Among these techniques, XPS, known for its high interfacial sensitivity, has become the primary tool for analyzing the chemical composition of SEI/CEI. AI has been applied to assist in the analysis of XPS data, significantly enhancing the efficiency and accuracy of interfacial studies. An ab initio framework based on a ML model was employed to predict the XPS results of SEI formed in LHCEs (Fig. 17e) [292]. The results demonstrated a high degree of consistency between the ML-predicted XPS outcomes and experimental results (Fig. 17f, g). This AI-driven prediction approach offers a novel solution for reducing the computational cost associated with XPS simulations.

**Fig. 18:**
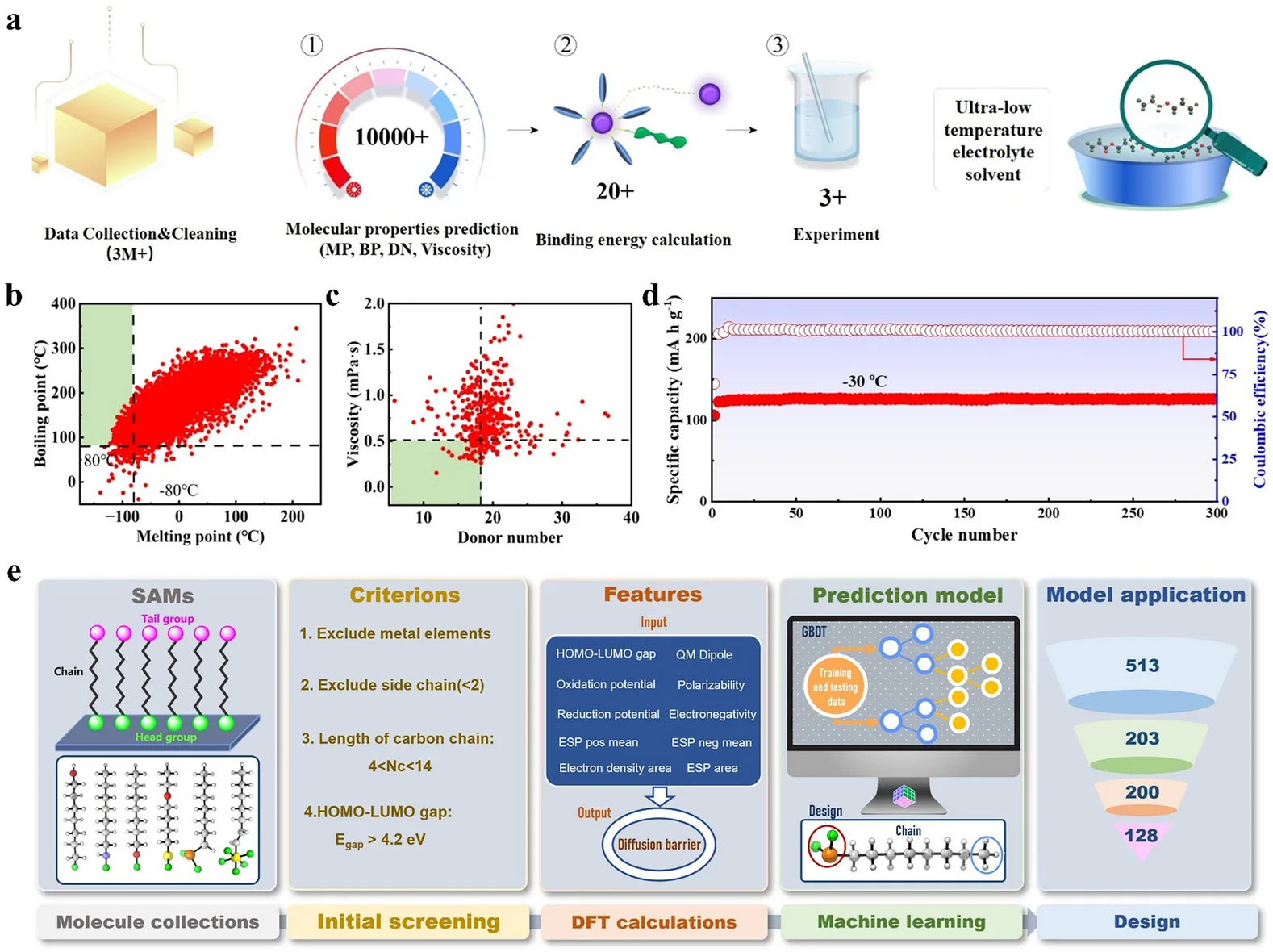
**a** Schematic diagram of the process for screening electrolyte molecules by combining machine learning and DFT [294]. The solvent diagram of **b** melting point versus boiling point data and **c** DN versus viscosity [294]. **d** Cycle performance of NCM811||Li cells using DPE electrolytes at 0.1 C and − 30 °C in the voltage window of 2.3–4.3 V [294]. **e** An overview of the data-driven material discovery workflow that has been employed in the current work for the identification of useful organic molecules for LMBs [295]

Zhang et al. proposed an efficient data–knowledge dual-driven framework for electrolyte molecule discovery, which integrates high-throughput computation, ML, and experimental validation [293]. They successfully identified three new carboxylic ester molecules–methyl trimethylacetate, ethyl trimethylacetate, and ethyl 2,2-dimethylbutanoate–from a candidate pool of 1,321,129 molecules. Compared with conventional carbonate-based electrolytes such as EC and DMC, the selected carboxylic ester electrolytes exhibited an anodic stability window exceeding 5.2 V and significantly enhanced reduction stability. This method demonstrates much higher screening efficiency and predictive accuracy than traditional trial-and-error approaches dominated by experimental testing, thereby greatly shortening the material development cycle and reducing research and development costs. Moreover, ML has also been combined with density functional theory (DFT) and other computational methods to fully leverage features such as solvation energy and molecular orbital energy levels. This integration enables more efficient prediction and screening of electrolyte solvents, Li salts, and functional additives. For example, Xiao et al. combined ML with DFT to design a temperature-resistant weakly solvating electrolyte capable of operating from − 70 to 70 °C (Fig. 18a) [294]. By predicting the MP, BP, viscosity, density, and DN of thousands of solvent molecules and calculating Li⁺ binding energies via DFT (Fig. 18b, c), they identified dipropyl ether (DPE) as the core solvent. The resulting electrolyte, prepared by dissolving 1.5 M LiFSI in DPE, forms anion-dominated solvation clusters that remain stable at low temperatures. Leveraging these advantages, the NCM811||Li coin cells using this electrolyte retained nearly 100% of their capacity after 300 cycles at − 30 °C (Fig. 18d). Additionally, an 8.5 Ah pouch cell retained 92.3% of its discharge capacity at − 70 °C and remained operational even at the extreme temperature of − 110 °C. Peng et al. combined DFT with a gradient boosting decision tree (GBDT) ML model to accurately predict self-assembled monolayers that can form artificial inorganic–organic hybrid interphases on LMAs, thereby enhancing cycling stability and mitigating dendrite growth (Fig. 18e) [295]. Specifically, they first extracted a series of structural descriptors representing molecular properties, which were then used as input features to train the GBDT model to capture the complex nonlinear relationship between molecular structure and Li⁺ diffusion barriers. By leveraging this model, they were able to rapidly screen and evaluate the potential performance of a large number of organic molecules, thus significantly reducing experimental time. Ultimately, from 128 candidate molecules sourced from the PubChem database, the research team identified eight optimal molecular structures exhibiting both low Li⁺ diffusion barriers and high mechanical stability. This approach not only demonstrates the remarkable potential of ML techniques in accelerating functional molecule screening and interfacial engineering but also provides new insights and tools for optimizing the interfacial stability of LMBs.

In summary, compared with conventional trial-and-error methods, AI-assisted approaches for designing LT electrolytes offer several distinct advantages: (1) traditional experimental methods typically require extensive material synthesis and testing, which are both time-consuming and costly. In contrast, machine learning can leverage existing experimental or simulation data to rapidly predict performance under different parameter combinations, significantly reducing the number of experiments and material consumption. (2) Traditional methods often rely on empirical models, which struggle to capture the underlying nonlinear relationships within complex material systems. In contrast, AI approaches can extract intricate, nonlinear structure–property relationships from high-dimensional data, thereby providing more accurate guidance for formulation optimization and mechanistic understanding. (3) Electrolyte design involves complex combinations of multiple salts, solvents, and additives, resulting in a vast parameter space. AI techniques can simultaneously handle high-dimensional variables and identify optimal regions that are often elusive to traditional approaches. (4) The predictive models developed by AI methods can be transferred to similar systems or extended to new material combinations, thereby reducing the need for extensive experimental validation in new systems. In contrast, traditional trial-and-error approaches typically require large-scale experiments for each newly developed system. Although AI demonstrates significant potential in screening and designing LT electrolytes, it still faces multiple challenges. Current AI-training datasets remain limited in scale and uneven in coverage, with the primary contributor to this limitation stemming from the lack of standardized experimental protocols across published studies. Consequently, establishing standardized experimental procedures and data analysis methodologies is of paramount importance for advancing AI applications in electrolyte development. Furthermore, the scarcity of key descriptors for characterizing electrolyte electrochemical performance presents a critical challenge. Determining how to accurately identify and define descriptors that effectively capture the LT characteristics of electrolytes remains an urgent issue requiring resolution during AI model construction.

## Summary and Future Perspectives

**Fig. 19:**
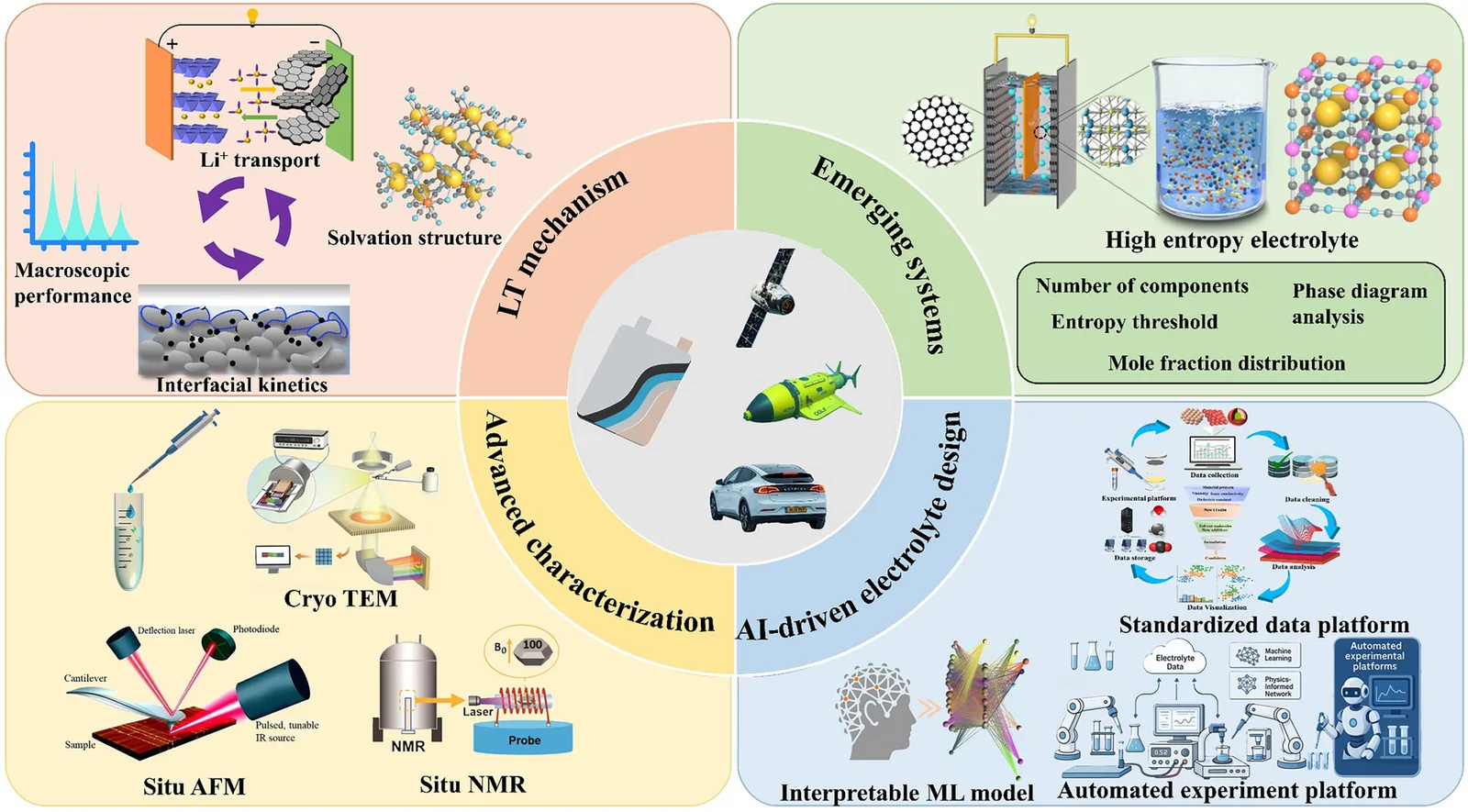
Development of LT electrolytes for LIBs

The limiting factors affecting the LT performance of LIBs are summarized in this study, including low ionic conductivity, sluggish charge transfer kinetics, slow Li⁺ diffusion across the SEI, and accelerated Li plating behavior. These limitations can result in severe capacity degradation, Li deposition, and increased safety risks. To address these challenges, optimization strategies for LT electrolytes are summarized. Advanced Li salts, solvents, film-forming additives, GPEs systems, and AI-assisted electrolyte design strategies are proposed to optimize the Li⁺ solvation structure, weaken Li⁺-solvent interactions, reduce desolvation energy barriers, and ultimately enhance the LT performance of LIBs. Although the research on LT LIBs has made significant progress in recent years, the research on the reaction mechanism of LIBs at LTs and high-performance electrolytes still needs to be further studied (Fig. 19).

1. The mechanisms underlying the various electrochemical reactions within batteries remain incompletely understood at LTs, particularly regarding interfacial reaction kinetics, Li⁺ desolvation behavior, and the evolution of SEI/CEI layers. For example, the transport of Li⁺ at the interface is not only limited by the structure of the SEI but is also closely linked to its solvation structure and desolvation energy barrier. At reduced temperatures, the energy barrier for Li⁺ to detach from its solvation shell increases markedly, and the SEI structure may undergo significant changes, posing challenges for elucidating the interfacial transport mechanism of Li⁺. Therefore, accurately elucidating the Li⁺ transport mechanism at low temperatures demands a multiscale and coupled research approach that integrates interfacial charge transfer and diffusion phenomena with a comprehensive understanding of the electrolyte's microscopic solvation structure and its macroscopic electrochemical behavior. At the same time, combining first-principles simulations can help capture key kinetic processes across different spatial and temporal scales, thereby enabling a deeper understanding of the multifaceted effects of LTs on interfacial reactions.
2. The development of novel electrolyte systems is a key strategy for enhancing the LT performance of LIBs. Among them, HEEs have emerged as a promising approach. These systems can reduce the Gibbs free energy and consequently depress the freezing point to levels comparable to that of single-solvent systems without increasing viscosity, thereby exhibiting excellent adaptability to LT environments. However, their definition and classification remain ambiguous and contentious. At present, there is no unified standard to define the entropy threshold, number of components, or compositional distribution that qualifies a system as “high entropy.” Moreover, a comprehensive set of descriptors capable of quantitatively correlating the structure and electrochemical performance of such systems is still lacking. Therefore, it is imperative to establish standardized criteria based on parameters such as component number, molar distribution, and entropy threshold to promote systematic research and development in this field. In HEE systems, the incorporation of diverse solvents, lithium salts, and functional additives leads to an exponential increase in the number of possible compositional combinations. This complexity significantly complicates the systematic screening and performance prediction of optimal formulations. Therefore, a critical challenge in current research lies in rapidly identifying synergistic component ratios within such highly complex mixtures. Constructing thermodynamic phase diagrams is considered a promising strategy to address this challenge. The key information provided by phase diagrams, such as component ratios, regions of thermodynamic stability, eutectic points, and phase transition temperatures, can serve as a valuable guide for the rational selection of HEE components. Moreover, the development of novel Li salts has emerged as a key research direction for enhancing the LT performance of LIBs. By precisely tailoring the molecular structure of Li salts, it becomes possible to simultaneously optimize ion dissociation degree, solvation structure, and interfacial film formation mechanisms. This approach not only contributes to improving ionic conductivity and interfacial stability under LT conditions but also helps balance the chemical stability and safety of electrolytes at sub-zero temperatures. As such, it represents an important future trend in the research and design of LT electrolytes for LIBs.
3. To further elucidate the complex chemical reaction mechanisms occurring within batteries at LTs, the development of advanced in situ and ex situ characterization techniques has become increasingly urgent. Techniques such as LT in situ nuclear magnetic resonance (NMR), in situ AFM, and cryogenic TEM (cryo-TEM) are particularly valuable. The combined use of NMR and Raman spectroscopy enables an in-depth analysis of solvation structures and Li^+^ transport mechanisms, thereby providing direct guidance for the optimization of salts, solvents, and additive systems. AFM and cryo-TEM offer multidimensional insights into the morphology, structure, mechanics, and chemistry of the SEI, enabling a deeper understanding of its formation and evolution. These tools provide critical means for designing structurally stable and compositionally controlled interfacial layers under LT conditions. Looking ahead, the multimodal integration and high-resolution advancement of characterization techniques will be vital for guiding electrolyte design and interface optimization under extreme temperature environments. As advanced characterization techniques generate increasingly complex datasets, future research should integrate data-driven approaches such as machine learning with these characterization data to enable automated feature extraction and performance prediction, thereby significantly enhancing the efficiency of LT electrolyte design.
4. The foundation of AI-assisted LT electrolyte design lies in the availability of electrolyte-related datasets. The quality, quantity, and reliability of these data are critical factors that determine the effectiveness of the designed electrolytes under low temperature. However, current datasets are primarily obtained through theoretical calculations and experimental measurements. Due to the lack of standardized research subjects, testing procedures, and characterization methods across the literature, significant inconsistencies exist among data collected from different studies. In the future, it is essential to establish large-scale, high-quality databases that encompass a wide range of LT electrolyte systems and testing conditions. At the same time, it is important to develop standardized experimental protocols, including electrochemical performance testing, interfacial characterization, and lifetime evaluation under LT conditions. These efforts will fundamentally improve the reusability and reliability of the data, thereby providing a solid foundation for AI-based modeling. The introduction of explainable techniques has effectively facilitated the application of AI in the design of LT electrolytes. However, the complex multi-component nature of electrolyte systems leads to highly nonlinear relationships among features, posing limitations for traditional interpretability methods. Therefore, it would be promising to develop physics-informed neural networks (PINNs) that incorporate electrochemical mechanisms and ion transport principles into the modeling process. Such models would rely not only on data but also on established physical laws to predict LT performance. This approach can enhance the interpretability of electrolyte formulation optimization and improve both transferability and credibility. For instance, LT electrolytes are required to deliver excellent performance under cold conditions while simultaneously ensuring high-temperature stability, safety, cost-effectiveness, and environmental compatibility. By embedding physical knowledge, PINNs can help researchers balance multiple performance indicators and engineering requirements, enabling the coordinated optimization of electrolyte systems that better meet practical application needs. The performance of LT electrolytes is influenced by multiple factors, including solvation structure, interfacial stability, viscosity, and ionic conductivity. In the future, integrating experimental measurements, MD simulations, and graph neural networks to couple macroscopic electrochemical properties with microscopic molecular structural features may enable the construction of multiscale models, thereby guiding the more precise design of LT electrolytes. For example, ML combined with MD simulations can be employed to predict the structure and stability of Li^+^ solvation shells at LTs under different electrolyte compositions and concentrations, thereby identifying formulations that facilitate rapid desolvation. In addition, it is possible to predict the decomposition products of electrolyte components during LT cycling, along with the composition, structure, and ion transport properties of the resulting SEI/CEI layers. This can guide the design of electrolytes that form interfacial films with high ionic conductivity and mechanical stability at LTs. Furthermore, by integrating electrochemical models with AI, it becomes feasible to predict Li plating tendencies under various LT conditions and optimize electrolyte formulations to suppress Li deposition. Moreover, the application of AI in the field of LT electrolytes remains largely confined to theoretical performance prediction and molecular screening, with most achievements limited to simulation and computational stages. Only a small fraction of these predictions has been experimentally validated, which poses a significant challenge to the transition from laboratory research to practical engineering applications. The rapid advancement of automated experimental platforms and AI-driven high-throughput computation technologies is offering robust technical support for the efficient screening and performance optimization of LT electrolytes. These automated-synthesis and testing platforms—integrating robotic control, precision liquid dispensing, high-sensitivity electrochemical measurements, and real-time data acquisition systems—can rapidly prepare and evaluate a wide variety of electrolyte formulations, greatly improving experimental efficiency and accelerating the transformation of theoretical insights into practical solutions.
